# Milk-Derived EVs from Different Animal Sources: An Overview on Their Detection, Isolation and Pleiotropic Exerted Effects

**DOI:** 10.3390/ijms27041938

**Published:** 2026-02-18

**Authors:** Ludovica Di Fabrizio, Faiza Abbas, Daniele Lopez, Mariele Montanari, Maria Carmela Scatà, Francesco Grandoni, Samanta Mecocci, Katia Cappelli, Paola Lanuti, Claudia Maria Radu, Genny Del Zotto, Stefano Papa, Anna Donniacuo, Alessandra Martucciello, Barbara Canonico

**Affiliations:** 1 Department of Biomolecular Sciences, DISB, University of Urbino Carlo Bo, 61029 Urbino, Italy; l.difabrizio1@campus.uniurb.it (L.D.F.); f.abbas@campus.uniurb.it (F.A.); daniele.lopez@uniurb.it (D.L.); mariele.montanari@uniurb.it (M.M.); stefano.papa@uniurb.it (S.P.); 2 Research Centre for Animal Production and Aquaculture, CREA, 00015 Monterotondo, Italy; mariacarmela.scata@crea.gov.it (M.C.S.); francesco.grandoni@crea.gov.it (F.G.); 3 Department of Veterinary Medicine, University of Perugia, 06126 Perugia, Italy; samanta.mecocci@unipg.it (S.M.); katia.cappelli@unipg.it (K.C.); 4 Department of Medicine and Aging Sciences, University “G. d’Annunzio”, Chieti-Pescara, 66100 Chieti, Italy; p.lanuti@unich.it; 5 First Chair of Internal Medicine and Thrombotic and Hemorrhagic Diseases Unit, Department of Medicine, Padova University Hospital, 35128 Padova, Italy; claudiamaria.radu@unipd.it; 6 Department of Research and Diagnostics IRCCS Istituto Giannina Gaslini Genoa, 16132 Genova, Italy; gennydelzotto@gaslini.org; 7 National Reference Centre for Hygiene and Technology of Breeding and Buffalo Production, Istituto Zooprofilattico Sperimentale del Mezzogiorno, 84131 Salerno, Italy; anna.donniacuo@izsmportici.it (A.D.); alessandra.martucciello@izsmportici.it (A.M.)

**Keywords:** milk-derived EVs (mEVs), mammalian milk EVs, conventional and imaging flow cytometric detection, EV fluorescent markers, mAb cross reactivity, tetraspanin detection, drug delivery systems, food-derived EVs

## Abstract

Milk is a primary source of vital nutrients and bioactive components fundamental to the growth and development of both newborn animals and humans. Produced by economically significant livestock species (including cattle, buffaloes, goats, sheep and camels), milk is a complex matrix rich in caseins, vitamins, fats, and proteins. Beyond its classical nutritional profile, milk serves as a pivotal vehicle for milk-derived extracellular vesicles (mEVs). These specialized food-derived EVs (fEVs) exert pleiotropic effects that resonate with the One Health paradigm, linking animal well-being and human nutrition to broader ecosystem stability. mEVs offer unique advantages, such as high biocompatibility and gastrointestinal stability, also rendering them potential therapeutic tools as drug delivery systems. However, challenges remain regarding the standardization of mEVs and the variability of their molecular cargo. This review provides a comprehensive comparative analysis of mEVs across a diverse taxonomic range, including bovines, water buffaloes, yaks, camels, goats, pigs, horses, donkeys, and humans, highlighting their distinct functional signatures. Indeed, a critical issue in mEV research is the isolation process: recommendations to minimize contamination from milk fat globules and casein micelles (which can cover EV signals) are given. Finally, current detection methods and instrumentation, with a specific focus on advancing flow cytometry (FC) approaches are discussed. Key insights include the use of conventional FC (with fluorescence triggering, the necessity of rigorous controls and calibration, and the utility of bead-based assays to overcome resolution limits) and imaging flow cytometry (IFC). In both technical approaches, the application of different EV generic fluorescent markers and the strategic selection of tetraspanins (i.e., CD9, CD63, CD81), is mandatory: we emphasize that selecting the correct antibody clones and accounting for inter-species cross-reactivity are essential steps for ensuring the accuracy and reproducibility of mEV research across mammalian species.

## 1. Introduction

Milk is the primary source of vital nutrients and bioactive components essential for the growth and development of both newborn animals and humans. It is produced by several livestock species of significant economic importance, notably cattle, water buffaloes, goats, sheep, and camels. Together with its derivatives, milk is a complex matrix and a rich source of nutritional, growth and immunological factors including casein, vitamins, fats, carbohydrates, proteins, and extracellular vesicles (EVs) [1,2,3].

Small EVs were first isolated from bovine milk in 1973, when they were described as membrane-derived vesicles approximately 100 nm in diameter, originating from the apical plasma membrane of mammary epithelial cells [4].

Since that initial discovery of bovine milk-derived EVs (mEVs), several studies have confirmed the presence of mEVs across a wide range of domestic animal species, including goats [5], water buffaloes [6], donkeys [7], pigs [8], horses [9], camels [10], and yaks [11].

EVs are a heterogeneous group of membrane-enclosed vesicles, including exosomes, microvesicles and apoptotic bodies, which differ in size and biogenesis and cellular origins. Exosomes typically range from 30 to 150 nm (representing the currently MISEV entities called small EVs), microvesicles from 100 to 1000 nm, and apoptotic bodies from 50 to 5000 nm [12,13]. Moreover, exosomes have an endocytic origin and are released from multivesicular bodies, which fuse with the plasma membrane, leading to the release of intraluminal vesicles into the extracellular microenvironment. In contrast, microvesicles are formed by the outward budding of the cell surface membrane, whereas apoptotic bodies are shed from the membrane of cells undergoing apoptosis [14,15].

Beyond size and morphology, these categories differ significantly in their molecular cargo [16], reflecting specific biogenesis pathways and specialized physiological functions [17,18]. The overlapping physical characteristics between exosomes and microvesicles, coupled with the lack of specific markers that differentiate them, have made it challenging to study these two populations individually. Therefore, the Minimal Information for Studies of Extracellular Vesicles (MISEV2023) guidelines, published by the International Society for Extracellular Vesicles (ISEV), recommends using the generic term “extracellular vesicle”, unless authors can identify reliable subcellular markers in their experimental models. Alternatively, EV subtypes can be referred to using operational terms such as small EVs (<200 nm; sEVs) and large EVs (>200 nm, lEVs) [19].

EVs carry a diverse array of biomolecules including proteins, lipids, metabolites and genetic material such as DNA, mRNA and non-coding RNAs (ncRNAs). This molecular cargo is shielded from degradation by extracellular proteases and nucleases by the vesicle’s robust lipid bilayer [20]. Their cargo enables EVs to mediate intercellular communication and influence various physiological and pathological processes.

The biocompatibility of EVs and the protection they confer to their cargo allow these molecules to bypass biological barriers and adverse environments, such as the gastrointestinal (GI) tract, without damage, while preserving their functionality [21]. Cargo is packaged by the parent cell in a regulated manner, and emerging evidence suggests that both the concentration of released EVs and the specific composition of their cargo vary according to the physiological or activation state of the originating cell [22]. The biological significance of EVs lies in their role as mediators of intercellular communication, capable of modulating the functions of recipient cells [23]. This interaction with recipient cells can occur locally or at distant systemic sites, involves complex ligand-receptor signaling at the cell surface, or the direct fusion of the vesicle with the plasma membrane. In most instances, EVs are internalized via endocytic pathways, leading to the integration of EV membrane components into the recipient cell and the release of cargo into the cytoplasm or nucleus. Recently, it was demonstrated that a fraction of endocytosed EVs reaches the nuclear compartment via the VOR (VAP-A-ORP3-Rab7) complex-mediated docking of late endosomes to the outer nuclear membrane in the nucleoplasmic reticulum [24]. Generally, EV-cargo transfer can activate new signaling pathways and elicit significant functional alterations in the target cell [25,26].

### 1.1. To Dot the i’s and Cross the t’s in mEV Research

**Figure 1:**
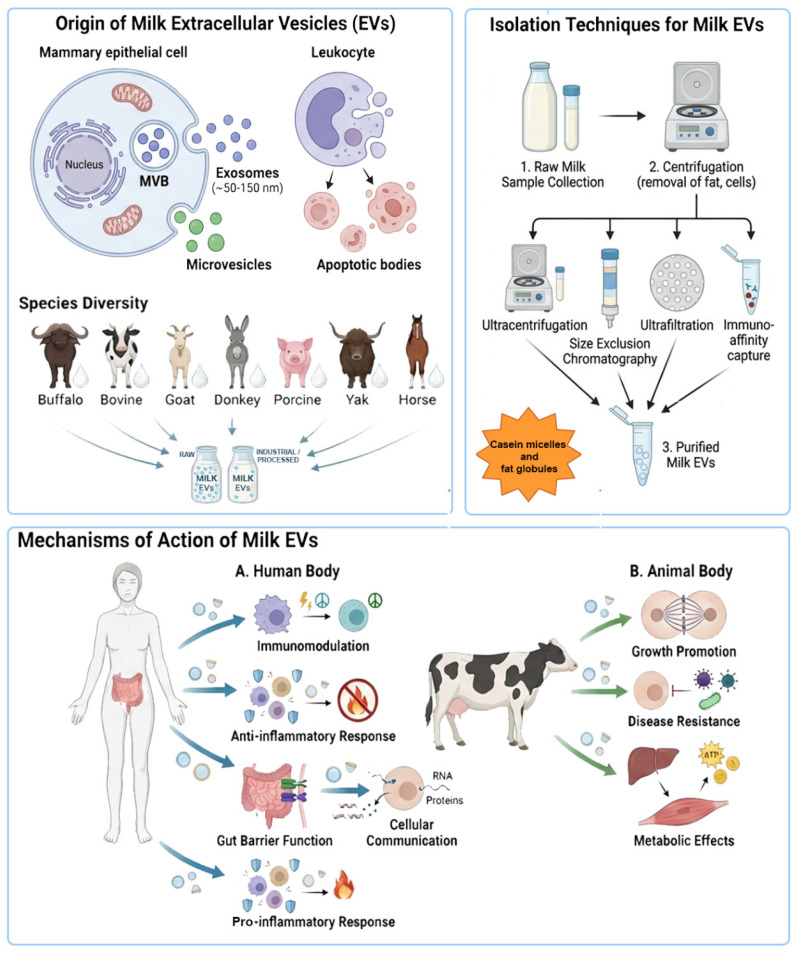
Summarizing figure of mEVs’ origin, isolation strategies, and proposed mechanisms of action. Figure adapted from an initial image generated by FigureLabs (figurelabs.ai) and manually modified by the authors.

The expanding interest in mEVs stems from their transformative therapeutic potential. Their inherent biocompatibility and unique ability to bypass biological and industrial hurdles—which often threaten conventional drug delivery systems—position them as a frontier in nanomedicine. However, translating mEVs from bench to bedside requires a paradigm shift toward high-precision detectability and rigorously validated protocols. A primary methodological “bottleneck” is the presence of casein micelles. With a size and density profile (100–200 nm) that closely mimics mEVs, their co-isolation is almost inevitable unless specifically verified [19]. Consequently, adhering to the latest MiSEV guidelines) [27] is not merely a recommendation but a necessity. In this context, verifying casein depletion [28] represents the quintessential “dotting of the i’s” for any robust isolation procedure (Figure 1) and will be cited in this review. Regarding mEV isolation and detection, mainly by FC, antibody validation is crucial to confirm that antibodies specifically bind to their target antigens without cross-reacting with unrelated proteins, however, given that the study of mEVs typically focuses on those found in dairy-producing livestock and related products rather than human milk-derived EVs (HmEVs), cross-reactivity becomes a strategic resource for labeling mEVs from diverse animal species using a limited set of monoclonal antibodies (mAbs) [29] (Figure 1). This approach effectively overcomes the scarcity of species-specific reagents for animals such as yaks, camels, or water buffalo [30,31]. Crucially, these technical considerations extend beyond mere methodology; they directly shape the biological activities of mEVs. While these effects are inherent to their origin, they are also significantly modulated by the specific isolation and labelling techniques employed [32]. Purification strategies can retain, alter, or strip away the “protein corona”, that rather than being considered a contamination element, may represent a functional determinant [33]. Therefore, the effects of mEVs are highly pleiotropic and can be even opposite (antagonistic), depending on the physiological context, the cargo of the vesicles, the recipient cell type, and methodologic features of the study. Indeed, although under conditions of inflammation or autoimmune disease, they could exacerbate pathogenic Th17 cell differentiation, potentially contributing to disease pathogenesis [1,34] while mEVs generally promote homeostasis in healthy individuals. This dualistic nature makes mEVs a “Janus-faced” tool in medicine, offering potential for both targeted therapy and the risk of unintended side effects (Figure 1). Furthermore, bovine mEVs can survive digestion [35,36], allowing their functional transfer (including membrane components or EV content) into the human body after consumption [37], but their quantity and quality cannot resist all industrial processing including pasteurization and filtration [38]. Importantly, the content and concentration of mEVs are affected by homogenization and the thermal processing of raw bovine milk. In alignment with the MISEV 2023 standards, the future of the field lies in orthogonal characterization. Relying on a single analytical method is no longer sufficient; instead, a suite of independent techniques must be employed to provide a comprehensive sample profile. Furthermore, the use of negative markers (to exclude casein micelles, fat globules, and lipoproteins) is now mandatory to unequivocally demonstrate the specific enrichment of EVs.

### 1.2. Pleiotropic Effects of mEVs Within the One Health Framework

The One Health paradigm acknowledges the intrinsic interconnectedness of human, animal, and environmental health. The study of mEVs provides a compelling illustration of this interconnectedness, as these vesicles act as cross-species biological messengers. Over the past decades, studies on EVs have become increasingly significant in life sciences due to their essential functions in both physiological and disease conditions. Almost all cells secrete different types of EVs, which can have a variety of effects. Recent studies have shown that mEVs appear to survive digestion and other harsh conditions; for this reason, they may help to protect or improve the intestinal barrier integrity or reduce inflammation in gut models [39,40,41]. mEVs also appear to have immunomodulatory properties as they can carry immunoregulatory miRNAs and can modulate macrophage differentiation, cytokine production, and inflammatory responses [1,42]. Beyond the immune system, mEVs have demonstrated significant osteogenic potential. For instance, the study conducted by Go et al. (2021) demonstrated how mEVs promote the differentiation and proliferation of Saos-2 cells by increasing the expression of the key osteogenic transcription factors RUNX2 and Osterix. A further study confirmed that mEVs could be used as anti-osteoporotic agents due to their ability to increase the number of osteocytes [43,44,45]. From a One Health perspective, the therapeutic potential of mEVs extends to veterinary applications. Recent findings suggest that mEVs from diverse sources, including colostrum, mature milk and milk from clinical mastitis, can counteract oxidative stress and ferroptosis in bovine mammary epithelial cells infected with *Klebsiella pneumoniae*, thereby providing a protective effect on mammary tissues [46]. Model limitations should be considered to avoid overinterpretation; specifically, most data originate from murine or cell culture studies [47,48,49], which may not translate to human physiology. The International Society for Extracellular Vesicles (ISEV) has established a Milk Task Force (https://www.isev.org/milk-task-force) accessed on 26 January 2020 [2,50,51] to provide standardized guidelines for reporting and conducting future experiments to address these translation gaps.

### 1.3. Advantages Offered by mEVs as Potential Therapeutic Tool

mEVs have gained significant attention due to their unique advantages, including their stability within the GI tract, biocompatibility, safety, and their substantial potential as vehicles for oral drug delivery [52,53]. The oral route is generally considered the most preferable method for drug administration; however, a significant number of therapeutic agents, including poorly water-soluble small molecules and macromolecular biologics such as peptides, exhibit low oral bioavailability. Although parenteral administration can overcome these limitations by enabling the rapid systemic delivery of unstable compounds, it remains an invasive procedure and is often associated with pain at the injection site and limited reversibility of drug effects [54,55].

Naturally optimized for the oral route, mEVs withstand gastric and enzymatic stress to reach the intestinal tract intact [56,57]. Their interaction with the gut environment is thought to modulate the gut–liver axis, an intricate physiological link mediated by the portal and systemic circulation. The synergy between the gut microbiome and the liver suggests that targeting the intestinal barrier via mEVs could offer a novel strategy for treating metabolic diseases. Consistently, mEVs exhibit greater mechanical rigidity and a more compact structure, ensuring stability in low-pH environments [58,59]. Following exposure to various conditions ranging from the mouth to the colon, mEVs remain intact, whereas the integrity of other synthetic nanoparticles, such as liposomes, is compromised [60]. Despite the resilience of mEVs in the GI tract, challenges remain in characterizing their absolute stability and bioavailability. Notably, Fonseca and colleagues [61] demonstrated that hydrophilic cargo often exhibits superior retention during digestion compared to lipophilic compounds, suggesting that mEVs may not serve as universal carriers for all therapeutic modalities. Beyond the GI tract, orally administered mEVs have been shown to distribute to various organs—including the liver, spleen, kidneys, pancreas, ovaries, lungs, heart, and brain—as evidenced by diverse fluorophore labelling studies [62,63].

In the context of gut disorders, where dysbiosis is a common pathological feature, mEVs exert therapeutic effects by regulating the abundance of gut microbiota and the excretion of bacterial EVs. For instance, oral administration of mEVs has been shown to restore intestinal immune homeostasis and microbiota balance, thereby alleviating conditions such as ulcerative colitis. Furthermore, mEVs significantly bolster intestinal health by promoting epithelial cell proliferation, supporting overall gut development, and mitigating apoptosis triggered by oxidative stress. Notably, these vesicles have been shown to modulate intestinal stem cell activity, specifically by upregulating the expression of key stemness markers. These activities are critical for maintaining intestinal epithelial homeostasis, a continuous process that is finely orchestrated by the balance between stem cell proliferation, migration, and differentiation, ensuring proper renewal and functional integrity of the gut lining [64,65]. Notably, milk-derived antimicrobial peptides (MAPs) found in EVs reduce infection risk in wounds by inhibiting the proliferation of pathogen strains such as *E. coli* and *S. aureus* [66].

### 1.4. Milk EVs as Food-Derived EVs

In recent years, food-derived EVs (fEVs) have garnered increasing attention due to emerging evidence linking diet to gut health, alongside the unique resilience of fEVs to the gastric environment. Among these, mEVs are particularly notable for their ability to survive ex vivo systems that mimic the harsh conditions of the GI tract [58]. mEVs have been proposed to modulate immune cells associated with the oral and gut mucosa while also supporting epithelial cell function, which is crucial for maintaining intestinal homeostasis [49]. Oral delivery remains the favored administration route due to its convenience, non-invasiveness, and cost-effectiveness. Beyond improving patient compliance, this route is physiologically significant; nanoparticulate systems, such as EVs, represent a promising strategy to overcome traditional oral delivery challenges. Specifically, EVs can encapsulate both hydrophilic and hydrophobic molecules, crossing biological barriers via specialized membrane-associated proteins [67,68,69].

**Figure 2:**
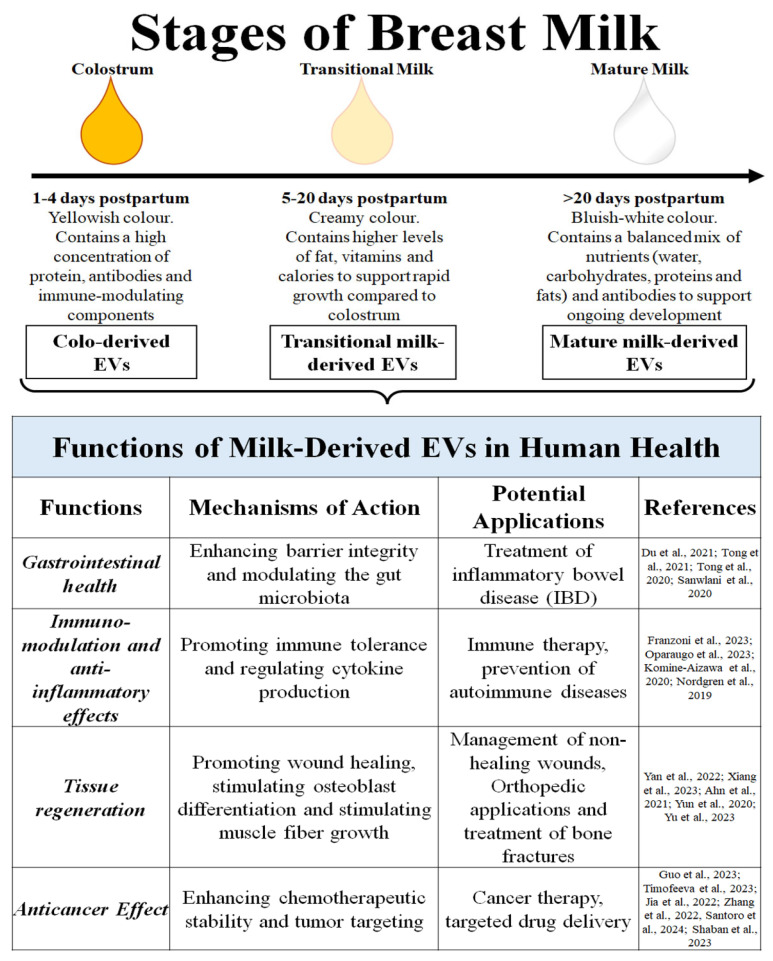
**Scheme of the different productions over the lactation curve:** features of the products and their specific EV content.

Significant attention has been directed toward the molecular cargo of milk exosomes, which encompasses a diverse array of proteins, lipids, and various RNA species, including mRNAs, miRNAs, circRNAs, and lncRNAs [70]. For instance, research on porcine mEVs demonstrated that vesicle-transported miRNAs safeguard the intestinal mucosa against lipopolysaccharide (LPS)-induced injury by inhibiting the NF-κB and p53 pathways. In particular, miR-148—the most prevalent miRNA in mEVs—has been shown to suppress the NF-κB signaling cascade, thereby mitigating colitis and its associated tumorigenesis [56,71]. The composition of these vesicles may vary as lactation progresses through three distinct physiological stages: colostrum (early postnatal), transitional milk (days 2–15), and mature milk (from one month post-calving) [72,73] (Figure 2).

Colostrum-derived EVs (ColoEVs) are enriched with bioactive molecules—including growth factors, proteins, miRNAs, and lipids—that facilitate a sophisticated signaling crosstalk between mother and offspring, essential for postnatal development [2]. Notably, EVs in colostrum are more abundant and possess higher protein concentration than those in mature milk; they also exhibit a more potent inhibitory effect on pro-inflammatory cytokines and apoptotic gene expression. In addition to their immunomodulatory roles, ColoEVs contribute to tissue repair by stimulating fibroblast proliferation and migration. Furthermore, ColoEVs show therapeutic potential in managing conditions such as mastitis, a common inflammatory disease of the mammary glands typically triggered by bacterial infections [74]. In addition, the characteristics of EVs derived from colostrum and mature milk differ in their capacity to mediate the function of a small intestine epithelial cell line (IEC-6 cells). This is crucial, as these cells play a vital role in forming the intestinal barrier and providing host defense against pathogens. These recent findings emphasize the potential of EVs from colostrum and mature milk to influence the health of various organs and systems including gut health, skin, and immune system [75]. From previous considerations, it is evident that several safety concerns exist when considering mEVs for human therapeutic use or as a nutritional supplement, similar to those associated with other cell-cultured milk technologies [76]. Primarily, the proteins carried within the mEVs could potentially elicit an immune response or allergic reactions in human consumers. Furthermore, there is a risk of contaminant enrichment, where residues from infections or drugs (e.g., antimicrobials) present in cattle milk samples could be concentrated within the vesicles themselves. Finally, given the rarity of studies suggesting a potential risk of increasing metastasis in certain cancer treatments, rigorous preclinical and clinical assessment is required to determine the specific mechanisms and long-term effects of mEVs in the human body [1].

### 1.5. Reported Issues for Dairy Animals mEVs and Their Cargo

Beyond general animal health, a critical challenge in mEV research lies in understanding how infectious and inflammatory pathologies alter milk composition and, by extension, EV yield and cargo. Although these dynamics have been extensively characterized in cattle, it is likely that similar mechanisms operate in goats and sheep, despite the limited literature currently available on this subject. In bovines, both clinical and subclinical mastitis profoundly disrupt milk yield and its biochemical profile—altering proteins, lipids, and inflammatory mediators—all of which may directly modulate EV biogenesis. Consequently, significant shifts in mEV concentration and miRNA signatures have been observed in animals suffering from mastitis or chronic viral infections, such as enzootic bovine leukosis. These findings underscore the potential of mEV profiles as sensitive biomarkers reflecting the inflammatory and immunological status of the mammary gland [77,78]. Comparable disease-related alterations are also documented in small ruminants. In dairy sheep, mastitis—both clinical and subclinical—induces marked changes in milk protein fractions, fat globule structure, and immune cell infiltration, which are known to affect the secretion of bioactive milk components [79]. Similarly, in goats, infectious diseases such as caprine arthritis encephalitis (CAE) and intramammary infections have been shown to modify milk yield, casein composition, and immune-related factors, potentially impacting the release and cargo of milk EVs [80]. Although direct evidence linking these conditions to mEV composition in goats and sheep is still scarce, the strong parallels with bovine mastitis strongly suggest that health-related variability of mEVs is a general phenomenon across dairy species rather than a cattle-specific issue. The limited number of omics-based studies available for sheep and goat milk EVs likely underestimates the magnitude of this problem. Comparative inter-species analyses confirm that transcriptomic and miRNA cargoes are highly sensitive to biological variables and the status of the host immune system [7]. These findings support the hypothesis that disease-driven modulation of EV cargo is a conserved physiological response across lactating mammals. Collectively, these observations underscore that animal health is a critical confounding factor when interpreting mEV data and must be considered equally across all dairy species. Neglecting inflammatory or infectious status may account for the high variability observed in the current literature, thereby hindering the identification of robust, biologically meaningful, and transferable EV-associated signatures In addition to health-related factors, breed-related variability represents a major biological source of heterogeneity in cattle mEVs. Dairy cattle have undergone intense genetic selection for divergent production traits, resulting in breeds that differ markedly in milk yield, fat and protein content, metabolic efficiency, and immune responsiveness. Such breed-specific physiological and metabolic differences are expected to influence not only bulk milk composition but also EV biogenesis, release dynamics, and cargo selection.

Evidence supporting this concept emerges from miRNomics and transcriptomic studies, which indicate that milk EV-associated miRNA and mRNA profiles vary according to genetic background. Breed-dependent differences in milk miRNA expression have been reported in cattle and other livestock species, with implications for immune regulation, metabolism, and mammary gland function [81]. Although cattle provide the most evident example due to the high number of specialized dairy breeds and the availability of molecular data, similar intra-species variability is likely to occur in other dairy animals such as goats and sheep, where fewer studies are currently available. This suggests that breed-related variability is a general issue in milk EV research rather than a species-specific anomaly.

Environmental and management-related factors further amplify this complexity. Diet composition, feeding strategies and nutrient availability are known to profoundly affect milk yield, lipid and protein fractions, and the metabolic status of lactating animals. These factors have also been shown to modulate EV-associated miRNA profiles, supporting a direct link between nutrition and EV cargo composition. Environmental stressors, including heat stress, housing conditions and seasonal variations, represent additional confounding variables. Heat stress in dairy cattle has been shown to alter milk exosomal miRNA expression patterns, particularly those involved in inflammation, stress response, and metabolic regulation [82]. Given the increasing impact of climate change on livestock systems, environment-driven modulation of mEV cargo is likely to become an increasingly relevant issue, especially when comparing studies conducted under different geographical and climatic conditions.

Understanding how genetic selection, environmental pressures, and animal welfare dictate the composition of mEVs is fundamental. This knowledge is essential not only for advancing basic biology but also for ensuring the safe and efficacious integration of mEVs into nutraceuticals, functional foods, and therapeutic platforms. Neglecting this multi-layered heterogeneity may undermine the reproducibility and translational potential of mEV research.

Beyond the safety risks stemming from animal health—such as bovine leukemia virus or mastitis—several critical challenges persist. These include intrinsic fluctuation in EV cargo, difficulties in isolation and scalability, and the rigorous demands of therapeutic application and industrial processing.

The molecular payload (comprising proteins, lipids, and transcriptomic signatures like miRNAs and mRNAs) and the overall mEV yield are highly susceptible to numerous variables [50]. Specifically, inter-breed differences significantly alter milk composition, thereby modifying inherent EV profiles. Furthermore, distinct features and the biochemical composition of mEVs are shaped by the lactation stage, dietary regimens, and broader environmental management practices.

***Bovine mEVs:***

Bovine milk has garnered significant scientific interest due to its high concentration of EVs. These vesicles have been reported to enhance cognitive function and support bone health; furthermore, their ability to protect cells from oxidative stress positions them as a promising tool in regenerative medicine [83]. Studies have also shown that such vesicles can reduce intestinal inflammation and support the gut microbiome [84,85]. Bovine mEVs can reduce inflammation by regulating nutrient metabolites such as increasing lipid anti-inflammatory metabolites and decreasing fecal amino acids; hence, they can be used to treat colitis [86]. Research findings highlighted that miRNAs derived from EVs are highly conserved in human, bovine, and caprine milk. Moreover, microRNAs such as miR-30a-5p, miR-22-3p, and miR-26a, which have a major role in regulating immune function, are commonly present in the colostrum and mature milk of cows and caprines [87]. Data suggest that cow mEVs contain TGFβ and miR-148a, which can regulate chondrocyte homeostasis and prevent cartilage damage; hence, they can be used as a therapeutic option for osteoarthritis [88]. Furthermore, investigations have shown that the oral delivery of bovine mEVs mitigates arthritis [89]. Experimental evidence suggests that bovine mEVs are highly biocompatible, as they can be readily absorbed by cells without producing cytotoxic effects; hence, they have emerged as a promising candidate for drug delivery applications in various therapeutic contexts [53].

***Water buffalo mEVs:***

Water buffalo (*Bubalus bubalis*) milk is characterized by higher levels of macronutrients compared to bovine milk, particularly regarding fat (6–8%) and protein (4–5%) content [90,91]. Additionally, the concentration of calcium is approximately 1.5 times higher than in cow’s milk [92]. Buffalo milk also contains antioxidant and anti-inflammatory compounds, such as delta-valerobetaine and biliverdin, alongside pentasaccharides and gangliosides, which are usually absent in cow’s milk [90]. In addition, specific probiotic species such as *Lactobacillus*, *Streptococcus*, *Lactococcus*, and *Enterococcus* are abundantly found in buffalo’s milk, which highlights its probiotic benefits [93]. Remarkably, buffalo milk contains a significant number of EVs. These EVs contain 96% of miRNA, which could help in immune regulation, blood vessel development, and epigenetic regulations [6]. Notably, a protein analysis conducted by Joshi et al. (2024) on EVs derived from three buffalo milk samples identified 331 common proteins. The biological functions of these proteins were related to immunity, cell cycle regulation, and metabolism. Significantly, the identification of 114 novel proteins in buffalo mEVs, when compared with bovine mEVs, suggests a crucial role in muscle development [94]. In 2023, Samuel et al. isolated and characterized EVs from cow, buffalo, sheep, and goat milk, which collectively account for 99% of global milk consumption. Comparative proteomic analysis of mEVs from these sources revealed that those from buffalo milk contained proteins potentially involved in immune regulation. Moreover, the study also determined the anti-cancer effects of buffalo mEVs, with data showing that these EVs induced a higher rate of cell death in colon cancer cells [3]. Further investigations highlighted that miRNAs obtained from the EVs of buffalo milk can play a pivotal role in both immune response and metabolism. Molecular profiling has also revealed 32 upregulated miRNAs in buffalo milk exosomes involved in metabolic pathways, while 16 downregulated miRNAs are anticipated to modulate immune responses [95]. Buffalo mEVs typically range from 50 to 200 nm in size. Comparative analyses between buffalo milk, plasma, and urine have shown that immune-related miRNAs (such as miR-21, miR-500, miR-125b, miR-155, and miR-27b) are particularly abundant in mEVs. Notably, miR-21 and miR-500 exhibit superior stability in milk compared to plasma or urine, underscoring the protective nature of the milk matrix [96].

***Yak mEVs:***

Yaks (*Bos grunniens*) living in high-altitude regions exhibit strong resistance to hypoxia and possess a high metabolic capacity. Small EVs (exosomes in the original paper) present in yak milk have been shown to activate hypoxia-inducible factor (HIF) signaling pathways, which promote the survival of intestinal epithelial cells (IECs) under conditions of oxygen deficiency. This mechanism plays a role in the yaks’ greater hypoxia tolerance compared with Holstein cows. Yak mEVs exhibited significantly higher expression of the proteins TSG101, CD63, and Hsp70 than cow milk-derived sEVs. Furthermore, yak mEVs were found to be more effective than cow mEVs in promoting the growth of IEC-6 cells under hypoxic conditions. Specifically, when the sEV concentration was between 200 and 240 ng/μL under hypoxic conditions, yak mEVs significantly increased IEC-6 cell survival post-treatment more effectively than cow mEVs [11,97].

***Goat mEVs:***

Goat mEVs have garnered significant scientific attention due to their unique properties and therapeutic potential across various pathologies. Studies indicate that goat mEVs possess potent immunomodulatory properties, suggesting their utility in treating autoimmune diseases and cancer [98]. Of note, experimental evidence revealed that goat mEVs possess higher loading capacity in comparison to cow and buffalo mEVs [99]. Moreover, EVs derived from goat milk can be applied as novel gene therapy vehicles, as they can effectively influence various metabolic pathways within recipient cells [71,100]. Investigations revealed that goat mEVs have potent antiviral activity and can significantly lower the infection caused by the dengue virus [101]. Since miRNAs play an important role in regulating the expression of many different genes in humans, thereby influencing cell differentiation and proliferation [102], a complete set of miRNAs was investigated in goat mEVs and the content was then compared and validated against miRNA EV content from cow milk. A total of 295 miRNAs were identified. Notably, goat milk samples contained a greater number of identified beta-miRNAs than cow milk. Moreover, miR-148a, miR-21-5p, miR-26a, and miR-30a-5p were found to be common between the two species [7,103].

***Camel mEVs:***

Camel milk has attracted scientific attention because of its distinctive composition and properties. Due to its anticancer, antibacterial, antidiabetic and immune-regulatory properties, it can be used as a biopharmaceutical agent. Interestingly, camel milk components can enhance reactive oxygen species (ROS) in cancer cells but reduce ROS in healthy cells. Its anticancer and immune-regulatory properties could be due to the lactoferrin and kappa casein mRNAs found within camel mEVs [104]. Furthermore, proteomic analysis revealed that camel mEVs were rich in proteins involved in EV synthesis and secretion processes including intracellular protein transport, translation and cell-to-cell adhesion [105]. In addition, investigations revealed that camel mEVs help in the reduction in colon damage caused by hypoxia; alongside this, they also enhance beneficial microbiota such as *Lactobacillus* and *Bifidobacterium* and reduce *Enterobacteriaceae*, suggesting their potential benefits as nutraceuticals and dietary supplements, especially for people residing in high altitudes [106]. Experimental evidence suggests that the oral administration of camel mEVs significantly reduced diet-induced obesity, caused by consuming a fat-rich diet, by increasing thermogenesis and regulating lipid metabolism. These unique vesicles decrease body fat percentage and lipid accumulation while simultaneously lowering the serum levels of triglycerides, free fatty acids, and cholesterol, thus providing a potential therapeutic option for treating obesity [107]. Camel mEVs can be utilized as novel carriers to transport curcumin, a polyphenol with chemo preventive and tumor-suppressive properties in lung cancer. In vitro antiproliferative assays demonstrated that curcumin delivered via camel mEVs significantly increased the cytotoxic effect against both drug-sensitive and taxol-resistant lung cancer cells when compared to free curcumin. These investigations suggest that camel mEVs represent an efficient carrier for the therapeutic delivery of curcumin [108]. Studies have shown that camel milk and its derived EVs reduce kidney damage and fibrosis while promoting oxidative balance in rats. Therefore, these vesicles can represent a promising therapeutic approach for the future treatment of diabetic nephropathy [109].

***Porcine mEVs:***

Porcine mEVs offer several health benefits, particularly concerning gut health and intestinal immunity. In vivo investigations in mice and piglets revealed that porcine mEVs enhanced the proportions of intestinal secretory immunoglobulin A (sIgA), which plays a significant role in gut-related immunity and mucosal homeostasis. Interestingly, an important circular RNA (circ-XPO4) present in porcine mEVs was found to enhance intestinal immunity. This enhancement is mediated by circ-XPO4 promoting the expression of the polymeric immunoglobulin receptor (pIgR) through the suppression of miR-221-5p in intestinal cells [110]. Ávila G. et al. (2025) investigated the effect of porcine mEVs on the proteome of porcine peripheral blood mononuclear cells (PBMCs). Gene Ontology (GO) enrichment analysis revealed that porcine mEV-treated cells were enriched in innate immunity-related proteins (i.e., TLR2, APOE, CD36, and MFGE8) compared to the control. In vitro experiments demonstrated that monocytes could internalize porcine mEVs, regulating immune functions by decreasing their phagocytic capacity and increasing their oxidative activity. Thus, these vesicles can act as immune regulators [111]. Interestingly, porcine mEVs can also be internalized by macrophages to deliver immune-relevant miRNAs. Moreover, such EVs promoted macrophage polarization toward the M2-like phenotype, consequently inducing anti-inflammatory effects [112]. Experimental evidence suggests that porcine mEVs play an important role in intestinal tract development. Data reported that these vesicles enhanced the proliferation of intestinal epithelial (IPEC-J2) cells by upregulating genes such as CDX2, IGF-1R, and PCNA, which are mainly involved in intestinal proliferation, consequently promoting digestive tract development [113]. Furthermore, researchers investigated mRNA and protein from porcine milk-sEVs for the first time. Transcriptomic analysis revealed a total of 16,304 mRNA; among these, 2409 were newly identified mRNAs, and some of them were involved in metabolism and signaling pathways. Proteomic analysis identified 639 proteins in total; most of them mainly resided in the cytoplasm and had a more specific role in immunity, while some of the proteins were tissue specific. Clusters of Orthologous Groups (COG) analysis revealed that many of the identified mRNAs and proteins were associated with cell cycle control and cell division [114]. Investigations suggested that porcine mEVs contain a high amount of miRNAs, which can transfer information from mother pig milk to piglets [115]. Around 1081 known and 2311 novel miRNAs were identified from pig mEVs. These miRNAs were known to be involved in cell signaling and development of the immune system [116].

***Horse mEVs:***

Experimental evidence suggested that horse mEVs contain around 5–8 different types of major proteins such as CD81, CD63 receptors, beta-lactoglobulin and lactadherin, actin, butyrophilin, lactoferrin, and xanthine dehydrogenase [117]. Research findings suggest that mare’s mEVs can be used as efficient drug delivery vehicles; investigations highlight that EVs loaded with quercetin efficiently improved β-galactosidase activity and cell viability in doxorubicin-treated cells and reduced damage to the myocardium, kidneys, and liver in aged model animals [9].

***Donkey mEVs:***

Caria et al. (2025) [118] performed the detailed characterization and proteomic analysis of EVs derived from donkey colostrum and mature donkey milk and found that EV constituents derived from both milks were involved in tissue repair and immunity. In particular, the proteins present in EVs from donkey colostrum play a role in defense and regeneration [112]. Donkey mEVs have shown anti-inflammatory properties, as they contain the amino acid asparagine, which can reduce intestinal injuries caused by bacterial lipopolysaccharides (LPSs). These EVs also possess strong antioxidant properties due to the presence of glutathione [119]. Detailed characterization of mRNA and the small RNA content of donkey mEVs also highlighted their immunomodulatory and anti-inflammatory potential; the role of donkey mEVs in lipid metabolism and reducing oxidative stress was also reported [7]. Liu et al. (2025) carried out the proteomic analysis of EVs from donkey colostrum and mature milk. Investigations revealed the presence of immune-associated proteins in colostrum; these proteins exhibited important roles in the autophagy and lysosome pathways, while EV proteins derived from mature milk were involved in the nutritional metabolism pathways [120]. All information described are summarized in Table 1.

***Human mEVs***

HmEVs act as natural nanocarriers, facilitating maternal–infant communication and delivering various functional “cargo” that remain largely intact through the infant’s digestive system [66]. The origin of HmEVs has recently propelled several research groups [125] to conduct in-depth investigations into mucus diffusivity, the endocytic mechanisms of epithelial uptake, and the composition of the human milk EV subpopulation transported across the intestinal epithelium, impacting the intestinal absorption of human milk EVs. HmEVs contain specific miRNAs that have been linked to the promotion of infant development and can potentially prevent certain diseases [126]. Clinical and epidemiological studies have confirmed the beneficial effects of human milk feeding (compared to infant formula), showing that it prevents both early and long-term diseases. Examples include necrotizing enterocolitis, neonatal sepsis, respiratory and GI tract infections, obesity, diabetes mellitus, allergic diseases, and malignancies [127,128,129]. HmEVs contribute to both short-term immunity and long-term developmental outcomes through a variety of mechanisms: (i) mEVs help modulate the infant’s developing immune system, promoting a balanced response, reducing excessive inflammation and potentially aiding in the management of conditions such as inflammatory bowel disease and colorectal cancer [64]. (ii) EVs play a significant role in the maturation and integrity of the infant’s gut. They promote the expression of tight junction proteins (like Claudin-1) and increase stem cell proliferation, making the gut lining stronger and less “leaky” [130], supporting gut barrier function and help establish a beneficial gut microbiota, particularly in preterm infants. (iii) The components within HmEVs exhibit antiviral effects against various pathogens, including HIV-1, rotavirus, and human cytomegalovirus (CMV) [131]. In detail, mEVs were demonstrated to promote the uptake of human immunodeficiency virus (HIV)-1 by macrophages and inhibited uptake by T cells [132]. Binding of EVs to antigen-presenting cells inhibited HIV infection of both dendritic cells and CD4+ T cells, suggesting mEVs as a novel protective factor against the vertical transmission of HIV-1, having the ability to attach to DC-SIGN receptors [133]; (iv) mEVs contain miRNAs that can contribute to the infant’s cognitive development, potentially leading to improved neurodevelopmental outcomes observed in breastfed children. Wijenayake and coworkers investigated the uptake of MEVs by human microglia cells in vitro and explored the functional outcomes of human mEV uptake, finding that EVs were taken up and localized in baseline and primed microglia. Indeed, this was the first evidence of mEV uptake by a brain macrophage, suggesting a potential role in regulating epigenetic machinery and neuroimmune modulation [134]. However, bovine EVs (bEVs) were also shown to pass the blood–brain barrier (BBB) of the pups, altering gene expression and promoting neuronal growth in the brain [135,136]. Based on the mentioned One Health approach, such findings seem to promote the concept of using EVs derived from human milk in infant feeding formulas to promote neurological function, however, this raises the ethical aspects of using human milk to derive EVs. (v) Finally, recent research suggests that HmEVs can enhance skeletal muscle growth and function [137] by impacting specific signaling pathways involved in muscle development. Parry et al. (2019) found that mEVs orchestrate intricate and context-dependent effects on skeletal muscle growth and maturation, although the investigation was for bovine EVs on mice [51,138]. Table 2 summarizes the diameter, concentration, and references with detailed pleiotropic effects of mEVs for the main, relevant reason that human research provides a wide range of reagents that can be used to explore various areas with effective, reliable application: the latter is an aspect that is far from obvious in the veterinary/One Health field, and finding suitable reagents requires additional tests and time.

Taken together, the findings of the in vitro, animal, and human evidence, the pleiotropic biological activities reported for mEVs across species—ranging from immunomodulation and gut barrier support to musculoskeletal and neurodevelopmental effects—strongly support their translational potential as nutraceuticals and therapeutic nanocarriers. In vitro studies have demonstrated that bovine and ***HmEVs*** reduce inflammatory cytokines (IL-17, IL-1β, IL-6), downregulate MMP9 expression, and suppress the NF-κB pathway while also enhancing intestinal barrier function by increasing tight junction proteins like claudin-1 and occludin [39,49,130]. Building on these findings, in vivo animal models have shown that bovine milk EVs can mitigate DSS-induced colitis by downregulating miR-125b [146], and ***HmEVs*** can reverse endothelial dysfunction in obese mice [47]. However, while these collective results highlight strong immunomodulatory and anti-inflammatory properties, human evidence remains limited as no clinical trials have yet been reported to confirm these therapeutic effects in patients. However, the strength of these functional claims is inherently linked to the analytical quality of the EV preparations used to generate them. Milk represents a uniquely complex matrix where vesicle-like contaminants (e.g., casein micelles, milk fat globules, lipoproteins and protein–RNA complexes) can co-isolate with EVs and, if not adequately removed, may confound both molecular profiling and functional readouts. Therefore, rigorous and milk-adapted isolation workflows are not a purely technical requirement but a prerequisite for interpreting biological effects with confidence and for enabling meaningful cross-study and cross-species comparisons.

### 1.6. Isolation Methods: Recommendations to Minimize Caseins and Fat Globules

The isolation of mEVs remains one of the most critical technical bottlenecks in the field, largely due to the intrinsic complexity of milk as a biological matrix. Different preparation strategies can markedly influence EV yield, purity, and subpopulation enrichment, ultimately affecting downstream molecular profiling and the interpretation of functional effects. For this reason, selecting an appropriate isolation workflow is essential not only to maximize recovery, but also to ensure that the observed biological activities are truly EV-associated rather than driven by co-isolated milk components. A wide range of EV isolation methods is currently available and frequently adopted in milk EV studies, each with its own set of advantages and limitations [147]. Minimal Information for Studies of Extracellular Vesicles (MISEV) guidelines include ultracentrifugation, density gradient centrifugation, exclusion chromatography, ultrafiltration, immunomagnetic separation, polymer precipitation, microfluidic platform, asymmetric flow field-flow fractionation, and anion exchange chromatography [19,148]. Crucially, these techniques exploit distinct physicochemical properties—such as size, density, solubility, or affinity—which explains why different methodologies enrich specific extracellular vesicle (EV) subsets while co-isolating diverse classes of contaminants. For instance, ultracentrifugation relies on sedimentation velocity and buoyancy, whereas polymer precipitation exploits solubility differences, and ultrafiltration operates strictly on size exclusion. While filtration, ultracentrifugation, and affinity-based capture remain the most prevalent isolation strategies, they must contend with complex sample matrices. The presence of various peptides, proteins, lipids, and cellular debris often mirror the structure of EVs or interact directly with them, complicating the extraction process and hindering purity [149,150]. Furthermore, we must remember that while general EV standards apply to the isolation of mEVs, specific measures must be observed. These are adjustments aligned with the MISEV guidelines, but they are currently undergoing further definition due to the more significant technical challenges involved. In fact, several studies have investigated the issue of contaminants, particularly fat globules and casein micelles, during the purification of EVs from milk samples. In this context, milk represents a particularly challenging biological matrix, as it is a complex colloidal system in which non-vesicular particles such as milk fat globules and casein micelles overlap with EVs in both size and buoyant density, thereby representing major sources of co-isolated contaminants [2]. One of the most critical sources of bias is contamination by milk fat globules and fat-derived particles. These lipid-rich structures can co-isolate with EVs during centrifugation-based procedures, particularly when pre-clearing steps are insufficient or when large sample volumes are processed. Residual lipid contamination may artificially inflate particle counts, interfere with size distribution analyses, and affect downstream functional assays.

Casein micelles constitute an additional and highly relevant confounding factor. Due to their size range and buoyant density, casein micelles frequently co-purify with small EVs, especially when ultracentrifugation is used as a single isolation step. Incomplete removal of caseins can result in protein aggregates or protein–RNA complexes that mimic EV-associated cargo, thereby complicating the interpretation of proteomic and transcriptomic data. In human milk, caseins (primarily the alpha, beta and kappa-isoforms) constitute a major portion of the protein content, accounting for approximately 3.6 g/L or about 40% of the total protein [151]. These proteins interact with colloidal calcium phosphate to form supramolecular assemblies known as casein micelles [152]. The size of casein micelles, typically ranging from 100 to 600 nm [153], overlaps with that of EVs, which span from <200 nm for small EVs to 200–1,000 nm for larger vesicles. Their buoyant density (around 1.06 g/mL) also closely matches the density range of EVs (1.08–1.19 g/mL) [151]. Because of these similarities, casein micelles frequently co-isolate with HmEVs, especially smaller EV subpopulations, regardless of the purification technique employed.

Consequently, casein removal or dissociation prior to EV isolation is a critical step to improve vesicle purity and analytical interpretability.

Similar considerations apply to bovine milk, where casein accounts for 80% of the total milk protein content to 35% in human breast milk [154], and where compositional differences may further influence casein–EV interactions and species-specific isolation challenges. In bovine milk, alpha-casein comprises more than half of the total casein fraction [155], whereas human milk is dominated by beta-casein, with approximate proportions at two weeks postpartum of 15% alpha-, 55% beta-, and 30% kappa-casein [156]. Alpha- and beta-casein also exhibit distinct biochemical characteristics: alpha-casein is more hydrophilic, whereas beta-casein has greater hydrophobicity [157].

These biochemical differences may influence casein–EV interactions and contribute to species-specific challenges in milk EV isolation and downstream molecular characterization. Accordingly, multiple pre-treatment strategies have been explored to reduce casein micelles and improve EV purity before downstream isolation. Cetinkaya et al. (2024) demonstrated that the combined use of chymosin and EDTA is highly effective and should be considered the preferred method for removing casein micelles from human milk samples [158]. Several studies have systematically compared different strategies to eliminate casein micelles, particularly in the context of EV isolation from bovine and porcine milk, highlighting that pre-treatment choice has a direct impact on EV purity and molecular yield. Rahman et al., 2019 evaluated the effects of acidification on mEVs with those of standard ultracentrifugation (UC), demonstrating that the acidification to pH 4.6 using HCl treatment significantly enhanced the isolation of bovine mEVs by effectively precipitating casein micelles. The authors concluded that acidification represents a rapid, cost-effective, and robust alternative to standard ultracentrifugation alone, providing high-purity vesicles suitable for advanced downstream applications such as RNA sequencing and functional assays [159]. Morphological analysis via TEM confirmed that acidified EVs retain their integrity, exhibiting the characteristic cup-shaped structure and typical size range (30–200 nm). Furthermore, the samples were found to be enriched significantly in exosomal markers (CD63, CD81) and RNA. The researchers concluded that acidification could represent a rapid, cost-effective, and superior alternative to standard UC, providing high-purity vesicles for advanced downstream applications like RNA sequencing and functional assays [146]. Wang et al. (2024) evaluated several pretreatment methods to overcome the common problem of casein contamination during the isolation of porcine mEVs. Their results showed that hydrochloric acid treatment (HA) before ultracentrifugation effectively removes caseins and other protein complexes. Compared to conventional ultracentrifugation (UC) alone or other chemical treatments, the pretreatment with HA followed by UC produced mEVs with significantly higher purity and fewer non-vesicular protein contaminants. Furthermore, the HA/UC method resulted in a much higher concentration of miRNA (specifically miR-148a-3p) per milligram of protein. Importantly, mEVs isolated using HA/UC also demonstrated superior biological performance in vitro, including enhanced cell proliferation and anti-inflammatory effects, reinforcing the functional relevance of effective casein depletion strategies [160].

In addition to contamination by casein micelles and milk fat globules, the recent literature highlights the frequent co-isolation of non-vesicular nanoparticles including lipoproteins and protein–RNA complexes, which cannot be reliably distinguished from EVs by commonly used particle quantification techniques [50,161]. Furthermore, regarding chemical and enzymatic pre-treatments, recent studies have shown that the technological history of milk samples, including homogenization and pasteurization, can substantially influence both EV integrity and the extent of co-isolated milk proteins and lipids. In particular, homogenization has been associated with increased carryover of non-vesicular milk components, further supporting the need for stringent pre-treatment and cleanup steps when working with processed milk [162].

Soluble milk proteins and protein aggregates further contribute to non-vesicular carryover, particularly in polymer-based precipitation methods or workflows lacking orthogonal purification steps. These contaminants may lead to the overestimation of EV-associated proteins and nucleic acids and obscure the identification of vesicle-specific markers. Growing evidence, indeed, supports the use of orthogonal isolation workflows, in which milk-specific pre-treatment steps are combined with downstream separation approaches such as size-exclusion chromatography or density-based methods. These multi-step strategies improve the analytical quality and reproducibility of mEV preparations compared with single-step ultracentrifugation [163,164]. At the same time, the lack of specific mEV markers and limited use of appropriate negative controls further contribute to uncertainty in EV identification and functional attribution.

Importantly, the presence of such contaminants does not only affect EV purity but also directly impacts EV characterization including nanoparticle tracking analysis, electron microscopy, and molecular profiling. As a result, differences in isolation protocols can generate substantial variability in reported EV concentration, size, and cargo composition across studies, even when similar starting materials are used.

Taken together, these methodological considerations underscore that differences in pre-analytical handling, pre-treatment choice, and purification strategy can substantially alter EV yield, purity, and cargo composition, ultimately shaping downstream analytical outputs and functional interpretations. Accordingly, detection strategies must be selected to (i) verify vesicle identity, (ii) quantify residual non-vesicular particles, and (iii) support reproducible links between EV molecular profiles and biological functions. This interdependence highlights the importance of combining orthogonal isolation steps with orthogonal detection methods that collectively validate EV integrity, purity, and functional relevance; indeed, transparent reporting of potential contaminants is essential to distinguish vesicle-associated signals from matrix-derived artifact.

### 1.7. Mainstream Technologies for EV Identification and Quantification

**Figure 3:**
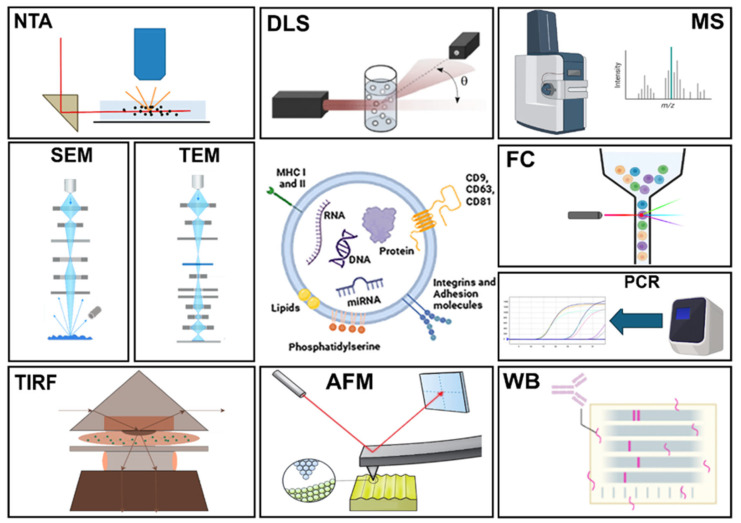
Representation of the different techniques involved in the evaluation of EVs: nanoparticle tracking analysis (NTA), dynamic light scattering (DLS), mass spectrometry (MS), scanning electron microscopy (SEM), transmission electron microscopy (TEM), flow cytometry (FC), polymerase chain reaction (PCR), total internal reflection fluorescence (TIRF), atomic force microscopy (AFM), and Western blotting (WB).

The investigation of EVs presents significant hurdles for the scientific community. Because each isolation strategy may selectively enrich distinct vesicle subpopulations and co-isolated components, robust characterization approaches are essential to distinguish EV-associated features from method-dependent artifacts and to ensure meaningful biological interpretation. Nevertheless, researchers studying EVs, focusing on their functional, physical and biochemical attributes, frequently encounter issues with reproducibility. A broad range of analytical tools is currently employed to detect EVs (Figure 3) such as nanoparticle tracking (NTA), atomic force microscopy (AFM), scanning electron microscopy (SEM), transmission electron microscopy (TEM), dynamic light scattering (DLS), mass spectrometry (MS), polymerase chain reaction (PCR), flow cytometry (FC), Western blot (WB), and total internal reflection fluorescence (TIRF) [165,166]. Each of these methods is able to obtain different information about the samples analyzed.

The NTA process involves two steps. First, particles in suspension are illuminated with a laser beam. Then, the scattered light is recorded using a light microscope. The mean square displacement of each particle is determined by tracking the Brownian motion of each particle. NTA quantifies the total number of particles, not EVs, thus, although it is a very valid method for measuring the EV size and concentration, it should be interpreted together with orthogonal confirmation of vesicle identity (e.g., TEM/cryo-TEM, FC, or marker-based assays) [167].

DLS, also known as photon correlation spectroscopy, is a technique that also depends on the scattering of a laser beam. In this technique, a monochromatic, coherent laser beam passes through a suspension of particles. If a particle happens to be in the beam’s path, the light is dispersed and scattered in all directions. By recording the intensity of the scattered light over time, the fluctuations due to the Brownian motion of the suspended particles can be observed. During these fluctuations, the distance between the scattered light beams changes constantly over time, leading to interference that is visible as minima (destructive interference) or maxima (enhanced interference) in the recorded spectrum. To obtain a particle size distribution, the autocorrelation function of the intensity spectra is generated and used for size determination [168,169]. A critical limitation is that DLS is highly weighted toward larger particles and may overestimate particle size or broaden distributions, and is best used in combination with imaging-based approaches.

MS is a powerful analytical technique that ionizes compounds and sorts them based on their mass-to-charge ratio. MS has been instrumental in the proteomic analysis of EVs, with advances in chromatography-coupled MS enhancing the identification of new EV protein biomarkers. The proteomic study of EVs usually involves three steps: isolating and purifying EVs; identifying proteins through MS; and analyzing the data in detail [170,171]. SEM focuses on surface morphology by utilizing secondary electron signals. A focused electron beam scans the sample surface, inducing the emission of secondary electrons, which are then collected by a specialized detector. These electrical signals then generate an image on a screen [172].

In TEM, an image is created by electron interference when the electron beam crosses the sample. Since the wavelength of the electron beam is shorter than the wavelength of visible light by three orders of magnitude, the images are recorded with a resolution of 1 nm. Unfortunately, the benefits from high resolution can be easily outweighed by disadvantages related to the measurement conditions and sample preparation. The specimens analyzed by TEM have to be fixed and dehydrated before the measurement. Additionally, the image acquisition is carried out under vacuum conditions. Nonetheless, electron microscopy is valuable for confirming vesicle-like structures, and the obtained images are then used for diameter determination in EV studies [173,174].

The PCR is widely recognized for its ability to detect nucleic acids including analyzing the varied compositions of nucleic acids found within EVs. Initial research utilizing techniques such as RT-qPCR and microarrays verified the presence of different types of RNA, including mRNA, miRNA and long non-coding RNA (lncRNA), within EVs [175]. In milk, protein–RNA complexes and non-vesicular carriers can contribute RNA signals that persist even when EV purity is suboptimal, potentially leading to an overestimation of EV cargo content. For stronger attribution of RNA to vesicles, studies should include RNase protection assays (±detergent) and report controls that exclude free RNA or RNA bound to non-vesicular complexes.

TIRF analysis is a sophisticated method of observing single molecules or nanoparticles. It involves monitoring their fluorescence following excitation by total internal reflection. This method enables accurate quantification through the counting of fluorescence spots and the measurement of their intensity [176].

MS is a powerful analytical technique that ionizes compounds and sorts them based on their mass-to-charge ratio. MS has been instrumental in the proteomic analysis of EVs, with advances in chromatography-coupled MS enhancing the identification of new EV protein biomarkers. The proteomic study of EVs usually involves three steps: isolating and purifying EVs; identifying proteins through MS; and analyzing the data in detail [170,171]. MS datasets may partly reflect co-isolated milk protein carryover rather than vesicle-specific cargo. Interpretation is strengthened by combining MS with enrichment strategies that reduce casein/fat globules upstream and by reporting both EV markers and likely contaminants to contextualize findings.

AFM is a technique that detects and records the interactions between a probing tip and a sample’s surface. A key feature of AFM is its ability to measure samples in their native state with minimal preparation. In this case, the EVs first had to be immobilized on a freshly cleaved mica surface and then scanned. AFM enables the acquisition of real 3D images of surface topography with very high resolution; however, for imaging to be successful, all vesicles must be attached to atomically flat surfaces such as mica [177].

WB is also important for profiling EV proteins. The process involves treating purified EVs with a buffer containing denaturants and protease inhibitors, followed by protein separation using sodium dodecyl sulfate polyacrylamide gel electrophoresis (SDS-PAGE). The proteins are then transferred to a cellulose membrane where they can be detected using specific antibodies and enhanced chemiluminescence [178,179]. WB interpretation benefits from: (i) positive EV markers, (ii) negative markers (to demonstrate depletion of non-EV cellular material), and (iii) explicit reporting of abundant milk proteins/caseins to document potential carryover. Similarly, ELISA-based detection may be impacted by non-specific adsorption or excess matrix proteins if cleanup is inadequate.

Importantly, each characterization method captures a distinct “dimension” of the preparation—such as size distribution, morphology, protein markers, or nucleic acid cargo—and exhibits differential sensitivity to milk-specific confounders. For example, light-scattering-based particle sizing and counting may be inflated by lipid droplets or protein aggregates, whereas protein- or fluorescence-based assays require stringent controls to exclude antibody/dye aggregates and non-vesicular carriers. Therefore, the most informative approach is typically a multi-platform workflow that integrates quantitative, imaging and molecular readouts to triangulate EV identity and purity. Among available tools, FC occupies a distinctive position because it can interrogate vesicles at the single-particle level (or via bead-assisted capture), enabling the assessment of surface markers and when appropriately controlled, the potential assignment of vesicle origin.

### 1.8. Insights into Flow Cytometry Approaches

**Figure 4:**
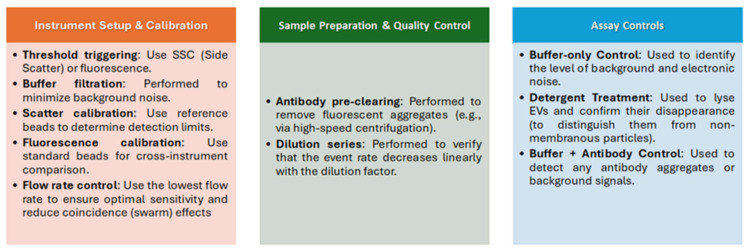
Schematic representation of the standardized framework for single EV detection and characterization by flow cytometry. Details reported by the MISEV guidelines.

FC is a technique that offers a multiparametric technology capable of identifying single EVs and measuring their cellular origin. It is typically employed in the characterization of EVs for biomarker expression. Flow cytometers measure fluorescence and light scattering signals originating from thousands of single particles per second in a fluid stream [180]. FC allows for the analysis and sorting of larger EVs and, through bead-based approaches, the capture of EVs labelled with fluorescent antibodies [28]. To overcome the sensitivity limits of conventional FC, several advanced strategies have been developed. Fluorescence triggering: By triggering the detection system on a bright fluorescence signal rather than light scattering, researchers can detect significantly smaller EVs This can be achieved using potent membrane dyes (e.g.,: lactadherin) or highly expressed markers. [181,182]. The Mechanism of Detection for Lactadherin (also known as MFG-E8) is based on C1 and C2 domains (contained in the molecule) that specifically interact with phosphatidylserine in a calcium-independent manner. For detection purposes, lactadherin is typically conjugated with fluorophores such as fluorescein isothiocyanate (FITC). The ISEV provides guidelines and works toward standardizing these measurements [183]. However, all of the other EV fluorescent markers can be used as the guide-parameter [181]; and these approaches, sometimes referred to as fluorescence-triggered flow cytometry (FT-FC) or single-EV flow cytometry (vFC), have become a standard strategy for rigorous and reproducible EV analysis. Following MISEV guidelines [184], extreme care must be taken to remove unbound dyes or dye aggregates, as these can be misidentified as EVs. Reliable data reporting requires controls and calibration. Rigorous controls (buffer-only, unstained samples, detergent-mediated EV lysis, and serial dilutions of samples or antibodies), together with the use of reference beads—particularly those that mimic the EV refractive index, such as hollow organosilica beads (HOBs) and polystyrene beads—are essential for generating reliable and reproducible data. A foundational set of recommendations was initially established in 2014, known as the Minimal Information for the Study of EVs (MISEV) [13], and was subsequently updated [185]. These guidelines should be followed in all aspects of EV research, from sample collection and preparation through to different technical approaches and final data reporting. The MIFlowCyt-EV standard provides specifications for the appropriate experimental design, reagents, protocols, and panels for EV characterization by FC. The FC analysis of EV presented several challenges (due to micro-sized particles) that are now gradually being resolved. Details are given in the following publications [184,186,187] and summarized in Figure 4. These controls serve as an indispensable reference point, facilitating comparisons across all other experimental samples and controls concerning critical parameters, such as the event rate, signal intensity, and the development of appropriate gating strategies, and properly define the boundaries separating true positive signals from instrumental or sample-related background noise.

### 1.9. Bead-Based Assay Multiplex

A common and broadly useful approach is to capture EVs onto larger, commercially available beads that are well within the detection range of conventional flow cytometers. This method allows for the phenotyping of EVs using specific antibodies, though it analyzes bulk populations rather than single EVs. Briefly, microbeads are coated with specific antibodies (e.g., anti-CD9, CD63, or CD81) or streptavidin (for biotinylated EVs) and are incubated with the sample to collect EVs [188]. Then, captured EVs are stained with fluorescently conjugated antibodies targeting surface proteins, and it is possible to perform the FC readout: the flow cytometer detects the fluorescence of the entire bead–EV complex. A higher fluorescent signal (as MFI) indicates a greater abundance of the target protein on the captured EVs. Different kits are commercially available such as multiplex bead assays (with fluorescently barcoded beads to simultaneously detect several different surface markers in a single sample) [142] and bead-conjugated EV assay detected (with high-affinity biotin–streptavidin interactions to capture EVs on 5 µm polystyrene beads for sensitive high-throughput clinical analysis). Furthermore, MISEV also furnishes details for bead-based EV detection [28], and this technique is largely employed [189].

### 1.10. Imaging Flow Cytometry (IFC)

IFC integrates the high-throughput capabilities of conventional FC, specifically the quantification of light scatter and multi-parametric fluorescence, with the morphological insights of high-resolution microscopy. Unlike traditional systems, IFC operates through a triggerless acquisition mechanism, characterized by zero dead time and continuous monitoring of the sample core, which effectively precludes coincidence-related data loss. The architectural features of IFC stem largely from the utilization of charge-coupled device (CCD) sensors rather than photomultiplier tubes (PMTs). CCDs offer several distinct analytical advantages: (1) effective signal-to-noise ratio, which significantly reduces electronic noise floor, (2) an enhanced linear dynamic range that guarantees greater flexibility in quantifying varying signal intensities, and (3) high quantum efficiency that leads to an optimal sensitivity for detecting low-abundance photons. IFC employs time delay integration (TDI) for CCD readout: by facilitating integration periods in the millisecond range, as opposed to the microsecond scales typical of PMT-based systems, TDI maximizes signal collection without incurring a readout noise penalty. Furthermore, every particle traversing the focal plane is recorded. This eliminates the requirement for a hardware trigger and minimizes the background interference Moreover, while coincidence events are inherently mitigated by the continuous flow architecture, the presence of multiple objects within a single frame does not compromise data quality. Such events are readily distinguished through automated image-based deconvolution or spatial filtering, allowing for the precise exclusion of artifacts from the final analytical dataset [190,191,192,193]. IFC methodologies have facilitated the longitudinal tracking of intracellular EV uptake and, more recently, the assessment of EV docking kinetics on the cell surface [194]. Nevertheless, the simultaneous profiling of multiple surface antigens on individual vesicles via IFC still presents substantial technical hurdles.

### 1.11. Pan-EV Fluorescent Probes

This category encompasses a variety of non-antibody-based fluorescent dyes and commercial kits designed to label and track EVs. These reagents target distinct structural components such as lipid membranes or luminal proteins. While antibody-mediated labeling remains a robust strategy for characterizing specific markers, its utility is often constrained by the low density of surface protein targets on certain EV subsets, which may fall below the detection threshold of many analytical platforms [195]. Furthermore, Loconte et al. demonstrated that the choice of labelling methodology significantly impacts the observation of EV–cell interactions [196]. This highlights the necessity of selecting the most suitable tracer—or ideally, a synergistic combination of probes—to accurately map internalization pathways. The following section provides a comprehensive overview of the most prevalent fluorescent probes, detailing their respective advantages and limitations to guide researchers in selecting the optimal tool for their specific applications.

1. Membrane dyes for single EV detection with and w/o EV separation: LCD (Lipophilic Cationic Dye) (LCD and FITC-conjugated phalloidin kit, BD Biosciences, San Jose, CA, USA) and vFRed™ (Cellarcus Biosciences, San Diego, CA, USA) belong to the group of dyes allowing for direct detection, sizing (with beads), and characterization (co-staining with antibodies) in complex samples like plasma, milk, or other body fluids. Membrane labeling with LCD is generally compatible with subsequent immunophenotyping, indeed, protocols utilizing LCD have been shown to provide repeatable and standardized counts of circulating EV sub-phenotypes [197]. LCD is lipophilic and positively charged, allowing it to intercalate into and “probe” the lipid bilayers of membrane-bearing structures like EVs, of note, when used with dyes like phalloidin (which binds to F-actin), LCD can distinguish intact EVs from damaged vesicles or cell debris. vFRed™ is a next-generation, far-red fluorescent lipophilic dye specifically engineered for the high-resolution labelling of EVs including small EVs and microvesicles. The labelling is achieved through the spontaneous insertion of the dye’s hydrophobic aliphatic chains into the phospholipid bilayer of the EVs [185,198]. AcoDyes (Acoerela, Singapore) are a patented series of highly water-soluble, fluorogenic membrane dyes from the company Acoerela designed for accurately tracking EVs both in vitro and in vivo. In fact, detection via FC can be performed without needing ultracentrifugation (UC). Specific dyes like Aco-490, Aco-430, and Aco-600 bind directly to EV membranes, differentiating true EVs from background noise: this depends on their chemistry, inducing the “light on” mechanism (i.e., different emission profiles for bound and unbound dyes). Therefore, all of these stainings can be used to label and detect EVs isolated by different techniques, besides the possibility of being employed for detection in biofluids, reducing the need for extensive enrichment steps that might alter vesicle morphology or count.
2. Membrane dye for single EV detection after EV separation: These lipophilic dyes incorporate into the lipid bilayer of the EVs; indeed, we want to underline that these dyes are employed on SEC-purified, ultracentrifuged, or differentially centrifuged EVs. Chen and coworkers found that PKH67 and PKH26 could maximally label ∼60–80% of EVs isolated from the conditioned cell culture medium [199]. Both PKH dyes are widely used but can form aggregates that interfere with detection, requiring careful use at optimal concentrations. Indeed, a density gradient centrifugation step (to remove unbound dye) is required by the manufacturer for the PKH67 protocol, although this step is inevitably associated with increased material loss during preparation. DiI and di-8-ANEPPS: These molecules can provide efficient and uniform labeling of EVs. Fluorescent dyes such as di-4-ANEPPS and di-8-ANEPPS are highly sensitive fluorescent dyes displaying consistent potentiometric responses in a wide variety of systems [200,201]. While they have similar spectral properties, di-8-ANEPPS is less water-soluble and more stable in the membrane than di-4-ANEPPS due to its longer hydrophobic carbon tails [202]. di-8-ANEPPS and high concentration of DiI could achieve the efficient and uniform labelling of EVs with nearly 100% labelling efficiency for di-8-ANEPPS and 70–100% for DiI in EVs isolated from the conditioned cell culture medium [199]. MemGlow and CellMask: These probes revealed a bright and sensitive staining of EV membranes with minimal aggregation, although MemGlow showed an affinity to VLDLs. In a recent study employing nanoFCM, these dyes (CellMask Deep Red, MemGlow 488 and 640) were compared with the following probes: ExoBrite 490/515 (Biotium, Fremont, CA, USA); ExoBrite 640/660 (Biotium); and CellTracker Deep Red (CTDR, Thermofisher, Waltham, MA, USA). The EV dilution and staining protocol is detailed by Brealey J and coworkers [195]. ExoBrite™ True EV Membrane Stains are offered as alternatives to traditional dyes and are noted for minimal background aggregation and high-resolution imaging performance, even by the spectral FC apparatus [203]. Furthermore, in IFC, a novel lipid dye called Exoria (Exopharm Limited, Melbourne, Australia) was recently applied [204]. Finally, calcein AM, calcein violet, and CFSE are additional generic markers for the FC detection of EVs in cell uptake studies [205]. Membrane labelling with CFSE [5-(and-6)-carboxyfluorescein diacetate succinimidyl ester] is normally performed at 37 °C, ensuring the optimal conditions for enzyme activity and thus the turnover to the fluorescent variant CFSE. However, Ender and coworkers (2020) [205] incubated EVs with 40 µM CFSE for 10 min at 4 °C or room temperature, finding the best yield of intact CFSE + EVs. In mEVs (from both bovine and human origin), the protein-binding dye CFSE was incubated at 37 °C for 2 h, at 40 μM concentration, and the free dye was removed by ultrafiltration at 2000 g for 30 min. Usually, this kind of labelling is carried out for cellular uptake experiments. De Rond et al. (2018) [206] reported an overview of the properties of different markers for plasma EVs including calcein violet and lactadherin, which had not been used as generic markers previously in their work [185], concluding that none of the generic markers detected all and only EVs. For milk EV specific cellular uptake experiments (and stability), calcein AM dye (Thermo Scientific) or CTDR were used following a well-established protocol, at a 10 µM concentration [207].

### 1.12. Tetraspanins CD9, CD63 and CD81 for Detecting mEVs

The organization of membrane microdomains, termed tetraspanin-enriched microdomains (TEMs), is performed by tetraspanins, a protein superfamily that form clusters and interact with a large variety of transmembrane and cytosolic signaling proteins [208]. CD9, CD63, and CD81 are among the tetraspanins that have a broad tissue distribution, while others such as CD37 and CD53 are restricted to particular tissues. Small EVs from MVB origin have been described as highly enriched in tetraspanins, and tetraspanins have been proposed as possible exosomal markers. Tetraspanins can interact with various receptors and signaling molecules at the membrane. Consequently, they may be involved in the attachment of sEVs to target cells and their absorption, or in antigen presentation in response to the immune system [209,210]. Research performed on the human cell line HEK293 analyzed the proteins most highly enriched in sEVs, identifying CD9, CD63, and CD81 as the most prevalent. These proteins have therefore been utilized for exosome/sEVs capture and detection. CD63 and CD81 are the most frequently identified proteins in exosomes and are considered classical markers [208,211]. As tetraspanins are expressed on the surface of EVs, they are extensively utilized in the flow cytometric analysis of said EVs. In immunoassays developed for EV phenotyping, the tetraspanins CD9, CD63, and CD81 are commonly used as bona fide EV-associated markers for total EV detection. These tetraspanins play a key role in EV formation, cargo selection/sorting, and EV release and uptake. Furthermore, the presence of specific combinations of CD9, CD63, and/or CD81 on EVs can provide information about their particular biogenesis pathway [212]. CD9 is a member of the tetraspanin family of proteins, which coordinates lateral interactions with other membrane proteins, particularly integrins, on the cell surface, playing a key role in essential cellular functions in many immune and endothelial cells. These functions include intracellular signaling, cell stimulation, and proliferation and cell viability. Subsequently, it became associated with numerous cellular processes including motility, proliferation, differentiation, fusion, and adhesion [213,214]. Some studies, such as those by Suzuki et al. (2009), have demonstrated that in a murine model, CD9 negatively regulates LPS-induced macrophage activation, and that knocking it out increases the infiltration of macrophages into the lungs [215]. Another study by Wang et al. (2002) showed that murine CD9 is expressed on all peritoneal macrophages but is downregulated upon activation by IFNγ [216]. Therefore, CD9 expression may be an indicator of antigen-presenting cell (APC) subsets with a higher capacity for T cell activation [213]. Milburn J.V. et al. (2021) conducted a study with the aim of using two novel mAbs to detect CD9 expression on porcine leukocytes. CD9 was found to be expressed on monocytes, indicating their role as antigen-presenting cells (APCs). The study demonstrated that CD9 is uniformly expressed on porcine monocytes, which are APC precursors, as well as on a distinct population of CD9+ porcine B cells. Therefore, it is possible that a CD9+ phenotype also represents a subset of porcine B cells with enhanced antigen presentation or T cell activation function. However, CD81 is the most widely used and informative marker, particularly for mEVs in all species [212,213].

### 1.13. Antibody Availability and Cross-Reactivity: Critical Issues in One Health EV Flow Cytometry

Unlike in humans, where a wide repertoire of antibodies recognizes homologous antigenic targets, one of the main limitations of veterinary FC is the reduced availability of species-specific mAbs. This limitation is particularly evident in non-model animal species, including water buffaloes, horses, and camels, in which monoclonals developed for phylogenetically related species, mainly cattle, are required. This approach exploits the ability of antibodies to cross-react with antigenic epitopes conserved across species. However, insufficient validation of antibody specificity in non-target species may result in non-specific binding and misleading signal attribution. This problem becomes most evident when these antibodies are used in the FC of EVs. Due to their small size, EVs have a low surface antigen density, resulting in a reduced signal-to-noise ratio and complicate antibody-based detection strategies. Therefore, the detection and characterization of EVs by FC requires careful clone selection, accurate antibody titration, and the use of appropriate technical and biological controls. The adherence to standardized guidelines, such as those proposed by ISEV and MIFlowCyt-EV, is particularly important to minimize artifacts and ensure reproducibility when cross-reactive antibodies are used.

Among the mAbs that cross-react with cattle, CD63, CD9, and CD81 are the most widely used markers in EV research. These proteins belong primarily to the tetraspanin family, which is conventionally employed for EV identification, together with components of the ESCRT machinery and members of the Rab protein family [217].

***CD9 clones***: The monoclonal antibody MM2/57 is a mouse IgG2b anti-CD9 antibody that recognizes a conserved CD9 epitope and has been reported to cross-react with bovine CD9. Due to this property, it has been used in FC, immunohistochemistry, and Western blotting across different species. Although peer-reviewed studies specifically validating this clone in cattle are limited, several reports indicate that MM2/57 and other anti-human CD9 antibodies can recognize the bovine homologue, supporting its use as a cross-reactive marker [218,219].

***CD81 and CD63:*** CD81 is commonly used as a canonical exosomal marker in both bovine and human milk EV studies, supported by the high sequence homology between the two species. For CD63, the mouse monoclonal antibody CC25 represents the most frequently used clone in bovine samples and has been reported to be effective in both FC and Western blotting. In contrast, other anti-human CD63 antibodies, such as H5C6, lack documented validation for cross-reactivity with bovine antigens and should therefore be applied with caution.

Overall, while a reliable panel of tetraspanin antibodies is available for bovine EV studies, the extension of these reagents to other veterinary species, including water buffalo, remains limited and generally requires dedicated experimental validation of cross-reactivity before routine application [220].

A bibliometric analysis was performed to identify the main patterns, trends, and perspectives on milk EVs. These analyses allowed us to identify the main themes, trends, and gaps in the literature. The search of documents on Web of Science as a reference database was built on specific keywords, namely “milk EVs” or “Yak Milk EVs”, or “Horse Milk EVs”, or “Buffalo Milk EVs”, or “Bovine Milk EVs”, or “Porcine Milk EVs”, or “Caprine Milk EVs”, or “Goat Milk EVs” or “Camel Milk EVs” and “Flow Cytometry” or “Fluorescence Detection” or “EV Tracers”, and “Tetraspanins” or “CD9”, or “CD81” or “CD63”, or “cross reactivity”, and “Gut health”, or “Immune Cell”, or “Bone Health”, or “Neuronal Development”.

**Figure 5:**
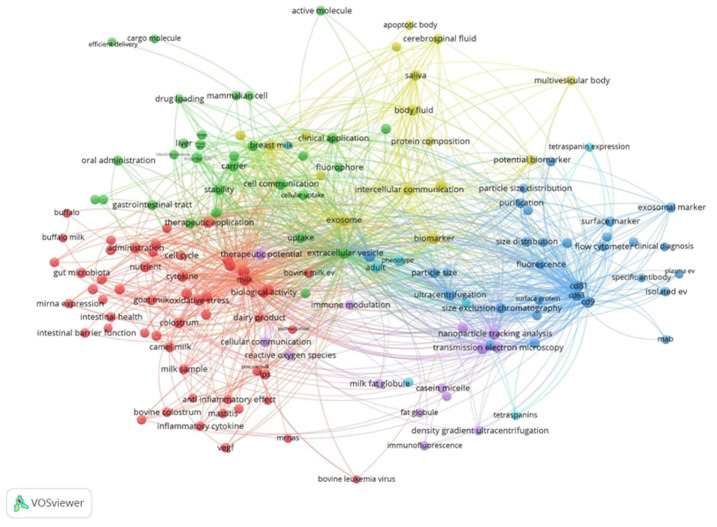
Keyword co-occurrence network generated through bibliometric analysis using VOSviewer: the figure illustrates the network of keywords extracted from selected publications in which the nodes represent keywords and their size is proportional to their frequency of occurrence. Links between nodes indicate co-occurrence relationships. Indeed, different colors denote thematic clusters that were automatically identified by the software. These clusters highlight the following: the lime cluster where terms are linked to EV characterization and biogenesis; the blue cluster where terms are related to tetraspanins and technical methods for isolating/analyzing EVs; the red cluster in which terms are related to inflammation, intestinal health, oxidative stress, and milk-derived components; the green cluster in which terms are associated with mEVs, GI tract uptake, and delivery applications, and finally, the purple cluster where terms are associated with milk proteins and structural components.

**Figure 6:**
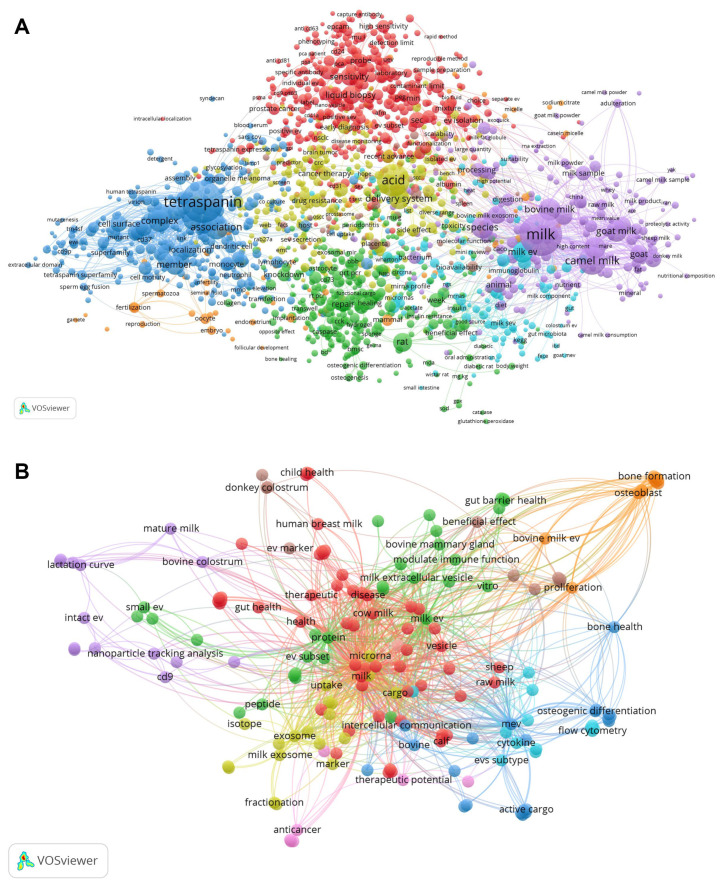
The VOSviewer analysis presented in both maps (**A**,**B**) illustrates the current research trends in milk EVs, highlighting various scientific investigations ranging from their fundamental characterization and biological mechanisms to their significant therapeutic potential in areas such as cancer treatment, bone formation, and gut health. (**A**) The red cluster focuses on the use of milk EVs in cancer treatment and related chemical/food processing topics. The green cluster reveals how EVs interact at a cellular level, their localization, and their potential as delivery systems. The blue cluster highlights specific biological components like tetraspanins, cell surface interactions, and cellular fusion processes. The purple cluster focuses on the origin of the milk (camel, bovine, goat) and immune-related components like immunoglobulins. (**B**) Orange cluster refers to osteogenic differentiation, bone formation processes, and the role of osteoblasts induced by milk EVs. Green cluster focuses on gut health induced by different sources of milk EVs. Red cluster highlights the ability of milk EVs to modulate the immune system and their use in treating various diseases. Purple/pink cluster concerns the active cargo within milk EVs with anticancer potential and the scientific methods used for analysis. Light blue cluster focuses on the characterization and analysis of EVs found in various types of raw milk. From the above presented VOSviewer analyses, it is possible to easily note that blue and purple clusters of Figure 6A are at both ends of the graph and, although few interconnections are visible, this distribution summarizes what is expressed several times in the main text, namely the difficulty of obtaining the reagents needed (mainly anti-tetraspanin antibodies) for the precise characterization of EVs in the milk of many animal species. In Figure 6B, this info is underlined, revealing CD9 (enclosed in the analyses of the purple/pink cluster) as the more recurrent antigen detected in colostrum and milk EVs.

A keyword co-occurrence analysis was performed via VOSviewer software (1.6.20 version) to generate network maps of the main research topics (i.e., clusters) and trends in keywords (Figure 5 and Figure 6).

Taken together, the wide array of detection and characterization approaches currently applied to mEVs highlights both the technological progress of the field and its intrinsic methodological fragility. Each analytical platform provides only a partial view of EV populations and is affected by specific biases related to sensitivity limits, co-isolated non-vesicular components, marker availability, and species-specific reagent performance. As a result, the apparent molecular composition and abundance of mEVs are strongly shaped by the combined effects of isolation and characterization strategies rather than reflecting an absolute biological entity.

## 2. Conclusions

This review highlights that in recent years, studies on mEVs have expanded to include several milk-producing mammalian species, focusing on their basic nutritional components and their potential as sophisticated mediators of communication between cells in the body, regardless of their species [1]. Admyre [140] first reported in 2007 that human breast milk is rich in mEVs, but the origin of these remains uncertain. They may originate from breast epithelial cells [221,222], macrophages, lymphocytes, or even cells from other parts of the body, which reach breast milk through the blood circulation. Similar to other body fluids, mEVs are composed of a variety of RNA, lipids, proteins, and so on. Several factors influence the content and biological activity of mEVs [223] such as gestational age, lactation period, maternal diet or nutrition, maternal disease, lifestyle, and stress. It also emerged that it is equally important to study mEVs from a One Health perspective. The health of humans, animals, and the environment they share is inextricably linked through the exchange of biological information, often mediated by EVs within the food chain and ecosystems. Recognizing EVs as mediators of this interspecies dialogue could provide a powerful tool for monitoring zoonotic threats, environmental stressors, and nutritional quality on a global scale. The immunomodulatory properties of cow, donkey, goat [7,85,86], and buffalo milk [3] and the unique hypoxia-resistance signals found in yak milk [11,97] demonstrate the profound interconnection between animal physiology and potential human therapeutic applications. Furthermore, the literature highlights the diverse challenges faced in EV characterization and isolation. As discussed in detail, the complexity of the milk matrix, particularly the presence of casein micelles and milk fat globules, requires standardized and rigorous isolation protocols to ensure EV purity and reproducibility. The analysis of detection methods highlights the importance of progress in FC and IFC. The selection of appropriate cellular markers and the critical assessment of cross-reactivity of mAbs between species are essential for the accurate characterization of mEVs in different species. Regarding the future application of mEVs, it is necessary to properly isolate and store them to maintain their biological activity. Consequently, the biological activities attributed to mEVs cannot be fully interpreted without considering the isolation approaches employed. Variability in EV composition arising from methodological choices may partially account for inconsistencies reported across functional studies. Taken together, the detection, isolation, and functional characterization of mEVs should be viewed as interdependent components of a single analytical framework rather than as independent steps. Adopting this integrated perspective is essential to correctly elucidate the biological effects and improve reproducibility across studies. Only through such rigorous standardization and a deeper understanding of the comparative biology of mEVs will we be able to fully exploit their potential as biocompatible, stable, and effective drug delivery systems or as functional dietary supplements. This will provide a reliable foundation for translational and One Health-oriented applications.

## Figures and Tables

**Table 1:** Summary of animal sources for breast milk/colostrum EVs: their diameter, concentrations, and cargo reported in specific references. Light green represents colostrum samples, green represents transitional milk, and dark green represents mature milk. (NA: Not Available).

Typology of Breast Milk	Animal Origins	Diameter	Concentration	Isolation Methods	Cargo (Proteins/miRNAs/mRNAs)	Reported Bioactivities	References
**Colostrum**	Holstein cows	149.6 (range of 88.5–239.0 nm)	4.3 × 10^11^ particles	Ultracentrifugation	Enriched in immunomodulatory proteins	Anti-inflammatory and protective effect against LPS -induced murine mastitis	[74]
	Bovine (Holstein cows), caprine (Saanen breed)	NA	NA	Serial differential centrifugations and Ultracentrifugation	Immune-related miRNAs i.e., miR-30a-5p, miR-22-3p, and miR-26a	Possible role in neonatal growth, development and immunity	[87]
	Donkey (Amiata breed)	153 ± 4.4 nm for DC-EVs	7.3 × 10^10^ (+9.3 × 10^9^)	Differential centrifugation/ Ultracentrifugation	Rich in proteins involved in specific defense functions, and regenerative processes.	Immunomodulatory, regenerative properties	[118]
	Bamei pigs and Landrace pigs	Sizes ranged from 50 to 100 nm	12.5 × 10^9^ for Bamei pigs and 17.5 × 10^9^ for Landrace pigs	Density gradient centrifugation	Enriched in immune-system-development associated miRNAs such as ssc-miR-143-5p, ssc-miR-7137-5p, and hsa-miR-3195.	Regulate the growth and immunity of piglets by activating relevant genes or pathways	[116]
	Bovine (Piemontese cows)	177.4 ± 2.4 nm	1.02 × 10^12^ (±4.88 × 10^10^)	Serial differential centrifugations and Ultracentrifugation	NA	Antimicrobial activity by downregulating cGAS/STING pathway responsible for inflammation and tissue damage	[40]
	Bovine	140 ± 5.1 nm	1.3 × 10^14^ ± 2.2 × 10^13^ particles/mL	Density gradient ultracentrifugation	NA	NA	[72]
	Buffalo	81 ± 26 nm	Average count: 318,539 particles	Serial differential centrifugations and Ultracentrifugation	bta-mir-2284o; bta-mir-301; bta-mir-12048; bta-mir-204; bta-mir-133c; bta-mir-365-1; bta-mir-18b; bta-mir-211; bta-mir-2285cp; bta-mir-181d	Enriched in miRNAs that regulate metabolic process of recipient cells, cell cycle, immune response and epigenetic regulation	[6]
	Ewe and goat	146.5 nm for ewe colostrum and 153.9 nm for goat colostrum	6.6 × 10^12^, for eve colostrum and 2.5 × 10^12^ for goat colostrum	Differential ultracentrifugation	NA	NA	[121]
	Porcine	140 nm	sEV 15 × 10^10^	Differential ultracentrifugation	High miRNAs (miR-146a-5p, miR-155-5p, and miR-22-3p)	Immunomodulatory function	[112]
**Transitional Milk or First Milk**	Bovine	156 ± 8.8 nm	9.3 × 10^13^ ± 1.8 × 10^13^ particles/mL	Density gradient ultracentrifugation	NA	NA	[72]
	Porcine	150 nm	sEV 9 × 10^10^	Differential ultracentrifugation	NA	NA	[112]
**Mature Milk**	Bovine	155.1 ± 16 nm	2.7 × 10^10^ ± 3 × 10^9^ particles mL^−1^	Differential ultracentrifugation	NA	NA	[122]
	Bovine	MM month 1 were 133 ± 2.6 nm, month 5 were 141 ± 1.0 nm and in month 9 were 136 ± 8.8 nm.	MM particle concentration month 1 was 2.3 × 10^12^ ± 1.5 × 10^12^ particles/mL, month 5 was 6.6 × 10^11^ ± 3.0 × 10^10^ particles/mL and month 9 was 3.9 × 10^12^ ± 1.6 × 10^12^ particles/mL	Density gradient ultracentrifugation	NA	NA	[72]
	Buffalo	60 ± 13 nm	Average count: 70,232 particles	Serial differential centrifugations and ultracentrifugation	bta-mir-103a-2; bta-mir-12031; bta-mir-487b; bta-mir-323; bta-mir-381; bta-mir-2285ap; bta-mir-12038; bta-mir-12061; bta-mir-2889; bta-mir-299; bta-mir-543; bta-mir-154b bta-mir-3956; bta-mir-6516: bta-mir-12010; bta-mir-154; bta-mir-329b; bta-mir-92b; bta-mir-485; bta-mir-412; bta-mir-453; bta-mir-493; bta-mir-495; bta-mir-380; bta-mir-411c; bta-mir-655; bta-mir-2284n; bta-mir-3578 bta-mir-136; bta-mir-758; bta-mir-323b; bta-mir-2285cl; bta-mir-379; bta-mir-541; bta-mir-654; bta-mir-409; bta-mir-127; bta-mir-2892; bta-mir-2397; bta-mir-376e; bta-mir-376d; bta-mir-154c; bta-mir-665; bta-mir-1185.	Rich in miRNAs involved in biological processes for cardiac, mitochondria and blood vessel development	[6]
	Cow, donkey, and goat	142.7 ± 2.9 nm for cow, 150.5 ± 3.2 for donkey, and 124.1 ± 2.3 for goat	1.22 × 10^12^ (±3.63 × 10^10^) for cow; 3.51 × 10^11^ (±1.22 × 10^10^) for donkey; and 7.39 × 10^11^ (±1.57 × 10^10^) for goat	Serial differential centrifugations and final ultracentrifugation	Messenger RNAs (mRNAs), Cow mEVs miRNAs (miR-340, miR181a, miR-362, miR-99a miR-532, miR-26b, miR-182 miR-429, miR-425, miR-34a). Donkey mEVs miRNAs (miR-374a, miR-152, miR-101, miR-98, miR-590, miR-320a, let-7d, miR23a, miR-145, let-7c). Goat mEVs miRNAs (miR-26a, let-7f, miR-27a, miR-22, miR-224, miR-16, miR-125b, miR-190a)	Enriched in key components involved in immune and inflammatory signals regulation. In particular, Goat and donkey mEVs were associated with transmembrane ion channels, gene transcription and epigenetic regulation. Donkey mEVs were involved in lipid metabolism and oxidative stress reduction	[7]
	Cows (Bos taurus) and yaks (Bos grunniens)	112.4 ± 48.6 nm	NA	Ultracentrifugation and ultracentrifugation with rennet precipitation	Enriched in specific components that could enhance hypoxia resistance in IEC-6 cells.	Yak mEVs activated the hypoxia-inducible factor signaling pathway in IEC-6, hence promoting higher hypoxia tolerance.	[11]
	Bovine leukemia virus-infected and uninfected cattle	milk sEVs from BLV-infected and uninfected cattle were 145.6 nm and 145.7 nm, respectively	milk sEVs from BLV-infected and uninfected cattle were 1.2 × 10^10^ and 4 × 10^10^, respectively	Ultracentrifugation	NA	NA	[123]
	Enzootic bovine leukosis-infected and uninfected cattle	Approximately 100 nm	NA	Ultracentrifugation	Eight mRNAs (TMEM156, SRGN, CXCL8, DEFB4A, FABP5, LAPTM5, LGALS1, and VIM) were significantly higher in milk sEVs of EBL cattle compared to uninfected cattle	Cattle at risk of EBL onset can be identified by testing the alteration in quantities of these **mRNAs biomarkers** in milk before they develop EBL.	[124]
	Murrah buffaloes	Range of 30–150 nm	NA	miRCURY exosome isolation kit	Enriched in proteins derived from extracellular origin and lysosomes	These proteins were mainly involved in immune response, metabolism and muscle development.	[94]
	BLV-uninfected healthy and EBL Holstein dairy cattle	100–150 nm	NA	Centrifugation and Ultracentrifugation	Higher levels of bta-miR-1246 and hsa-miR-424-5p in milk sEVs from EBL cattle compared to uninfected cattle	These two miRNAs could be possible biomarker for Enzootic bovine leukosis (EBL)	[78]
	Cow, buffalo, sheep, and goat	115, 105, 135 and 105 nm for the Cow, Buffalo, Goat and Sheep samples, respectively.	9 × 10^6^, 11 × 10^6^, 4.8 × 10^6^ and 4.2 × 10^6^ for the Cow, Buffalo, Goat and Sheep samples, respectively.	Differential centrifugation and ultracentrifugation	Buffalo and cow mEVs were enriched in immunomodulatory proteins.	Buffalo-milk-EVs induced significantly higher cell death in colon cancer cells.	[3]
	Donkey (Amiata breed)	143.4 ± 8.2 nm for MDM-EVs	3.7 × 10^10^ (+5.9 × 10^9^)	Differential centrifugation and ultracentrifugation	Highly expressed proteins: immunoglobulin binding, tertiary and ficolin-1-rich granule lumen transforming growth factor-beta-induced protein ig-h3 (TGFBI), extracellular matrix protein 2 (ECM2), collagen type XV alpha 1 chain (COL15A1), and cadherin-1 (CDH1), fatty acid binding protein 3 (FABP3), lipoprotein lipase (LPL), phosphoglucomutase-1 (PGM1).	Specific role in immune regulation, defensive, cell adhesion, and signaling interactions with host tissues (e.g., gut epithelium) and lipid metabolism respectively	[118]
	Bamei pigs and landrace pigs	Sizes ranged from 50 to 100 nm	11 × 10^9^ for Bamei colostrum and 12.5 × 10^9^ for Landrace pigs	Density gradient centrifugation	In Bamei pig mature milk EVs ssc-miR-205, ssc-miR-1296-5p, and ssc-miR-455-5p were upregulated compared to its colostrum. In landrace pigs’ mature milk EVs ssc-miR-128, ssc-miR-221-3p, and ssc-miR-222 were highly expressed compared to its colostrum.	These differentially expressed miRNAs are closely associated with processes such as cell signaling, cell physiology, and immune system development.	[116]
	Bovine and human	The size varied within the 30- to 200-nm range	NA	Serial differential centrifugations and ultracentrifugation	Enriched in intestinal barrier–related contents.	Restores gut barrier integrity and prevents endotoxin translocation into the liver consequently, reducing gut and liver associated inflammation.	[60]
	Camelus (C.) dromedarius, *C. bactrianus* and hybrids	25–170 nm	9.49 × 10^8^–4.18 × 10^10^	Density gradient ultracentrifugation	Packed with exosomal protein (CD9, CD63, CD81, HSP70, HSP90, TSG101 and ADAM10).	Exosome synthesis and its secretion processes i-e. intracellular protein transport and translation	[105]
	Porcine (Landrace pigs)	146.9 nm	NA	Fractional centrifugation and ultracentrifugation	Abundant amount of circRNA (circ-XPO4).	Enhanced expression level of intestinal secretory immunoglobulin A (SIgA) and the polymeric immunoglobulin receptor (pIgR) both in mice and piglet.	[110]
	Porcine	152 nm	sEV 4.8 × 10^10^	Differential ultracentrifugation	Packed with immune-related miRNAs such as miR-146a-5p, miR-155-5p, and miR-22-3p.	Promoted macrophage polarization toward the M2-like phenotype and employed anti-inflammatory effects in vitro.	[112]
	Mare horse	Different isolation methods: SEC (110 ± 8 nm), TEIK (118 ± 13 nm), and IP (131.5 ± 16 nm)	NA	Three isolation methods used: Total EV isolation by kit reagent; isoelectric precipitation; size exclusion chromatography	Encapsulated with drug (i.e., quercetin).	EVs loaded with quercetin significantly restored β-galactosidase activity and cellular viability in doxorubicin-treated cells, hence can be utilized as potential drug carriers.	[9]

**Table 2:** Summary of the specific human source (among Kingdom Animalia) for breast milk/colostrum EVs: their diameter, concentrations, and cargo reported in precise references. Light blue represents colostrum samples, blue represents transitional milk, and dark blue represents mature milk (NA: Not Available).

Typology of Breast Milk	Animal Origins	Diameter	Concentration	Isolation Methods	Cargo (Proteins/miRNAs/mRNAs)	Reported Bioactivities	References
**Colostrum**	Human	NA	NA	Serial differential centrifugations & Ultracentrifugation	Immune-related miRNAs i.e., miR-30a-5p, miR-22-3p, and miR-26a	Possible role in neonatal growth, development and immunity.	[87]
	Human	258.8 nm (mode: 196.1 ± 7.4 nm)	4.96 × 10^12^ ± 3.21 × 10^10^ particles/mL	Serial centrifugation/ExoQuick kit solution	NA	Antiviral activity against rotavirus and respiratory syncytial virus by inhibiting early steps of viral replication.	[139]
	Human	150.2 nm for human colostrum	2.1 × 10^13^ for human colostrum	Differential ultracentrifugation	NA	NA	[121]
	Human	c.a. 50 nm	NA	Ultracentrifugation and/or immuno-isolation on paramagnetic beads.	Higher expression of MHC class II, CD86, and the tetraspanin proteins CD63 and CD81	Inhibit anti-CD3-induced cytokine production from PBMC and increase the number of Foxp3+ CD4+ CD25+ T regulatory cells, subsequently influence the immune system of the infant.	[140]
**Transitional Milk or First Milk**	Human	188 nm for term and 161 nm for preterm human transitional milk	6 × 10^6^ for term and 8 × 10^6^ for preterm human transitional milk	Serial differential centrifugations and ultracentrifugation	MicroRNAs that could be a mediator of the anti-inflammatory effects,	Reduced IL-1β secretion and inflammasome-induced cell death.	[49]
	Human	10 to 210 nm	NA	Differential ultracentrifugation	NA	NA	[141]
**Mature Milk**	Human	103.8 nm (96–116.6 nm) for Fresh milk, 104.7 nm (101.7–113.3 nm) for Frozen milk	3.85 × 10^11^ (2.11–4.45 × 10^11^) for Fresh milk, 3.93 × 10^11^ (2.23–5.02 × 10^11^) for Frozen milk	Ultracentrifugation	miRNA-148a-3p (top conserved micoRNA)	NA	[142]
	Human	163.5 nm ± 83.5	1.5 × 10^11^ ± 3.4 × 10^10^ particles/mL	ExoEasy Maxi kit	Highly expressed miRNAs (miR-4271, miR-3197, and miR-2861)	Specific role in endocrine signaling, cellular community, and neurodevelopment.	[143]
	Human	150.4 ± 69.3 nm	7.08 × 10^8^ particles/mL	Ultracentrifugation	Proteins and miRNA (miRNA-29a) that suppress lipid synthesis and promote lipidolysis.	Efficiently reduced high fat diet-induced hepatic steatosis and insulin resistance in mice with NAFLD by inhibiting lipogenesis and increasing lipolysis.	[144]
	Human	The size varied within the 30- to 600-nm range	NA	Serial differential centrifugations and ultracentrifugation	Enriched in intestinal barrier–related components	Restores gut barrier integrity and prevents endotoxin translocation into the liver consequently, reducing gut and liver associated inflammation.	[60]
	Human	149.7 ± 20.7 nm	2.18 ± 1.53 × 10^11^	Ultracentrifugation	**Top expressed micro RNAs.** hsa-miR-30d-5p† hsa-let-7b-5p hsa-let-7a-5p hsa-miR-125a-5p† hsa-miR-21-5p hsa-miR-423-5p† hsa-let-7g-5p hsa-let-7f-5p hsa-miR-30a-5p hsa-miR-146b-5p	NA	[145]
	Human	The pellet contains 100–200 nm EVs, while the supernatant contains smaller vesicle-like particles (≤ 50 nm)	8.18 × 10^10^ particles/mL	Differential ultracentrifugation and serial filtration method	Highly expressed microRNAs (miR-148/152 family) that modulate DNMT1 and epigenetic machinery.	Uptake of EVs by a brain macrophage in vitro, suggesting their crucial role in regulating epigenetic machinery and neuroimmune modulation.	[134]

## Data Availability

No new data were created or analyzed in this study. Data sharing is not applicable to this article.
